# Bacterial Adaptive Responses to Green and Chemically Synthesized Silver Nanoparticles: Implications for Resistance Development

**DOI:** 10.3390/nano16120730

**Published:** 2026-06-12

**Authors:** Akamu J. Ewunkem, Joy T. Godbolt, Josiah Dixon, Jordan Queenie, Larisa C. Kiki, Monela Ntonifor, Uchenna Iloghalu

**Affiliations:** 1 Department of Biological Sciences, Winston Salem State University, Winston Salem, NC 27110, USA; jgodbolt122@rams.wssu.edu (J.T.G.); jdixon119@rams.wssu.edu (J.D.); jqueenie123@rams.wssu.edu (J.Q.); iloghaluub@wssu.edu (U.I.); 2 Applied Science & Technology, North Carolina A and T State University, Greensboro, NC 27411, USA; klchila@aggies.ncat.edu (L.C.K.); mmntonifor@aggies.ncat.edu (M.N.)

**Keywords:** silver nanoparticles, resistance, polymorphism, *E. coli*, experimental evolution

## Abstract

The misuse of antibiotics is causing widespread antibiotic resistance, creating an urgent need for new treatment options such as nanoparticle-based therapies. This study aimed to compare silver nanoparticles (AgNPs) produced via green synthesis methods with those made through traditional chemical processes. Furthermore, the study investigated and contrasted the bacterial responses to these two types of AgNPs over a 21-day period of selection pressure using experimental evolution techniques. Analysis using scanning electron microscopy and transmission electron microscopy revealed a consistent, uniform morphology among the AgNPs produced via chemical methods. In contrast, AgNPs synthesized through green methods displayed an irregular morphology. Despite these morphological differences, all nanoparticles from both synthesis approaches were under 100 nm in diameter. These findings were further supported by the absorption spectrum data, which showed a maximum absorption peak between the 400 and 500 nm wavelength range. *E. coli* exposed to green synthesized AgNPs for 21 days adapted to their presence, exhibiting both enhanced resistance to the green synthesized AgNPs themselves and the development of cross-resistance to ionic silver, a pattern not observed in chemically synthesized AgNP-selected populations. Populations selected using chemical synthesized AgNPs did not develop increased resistance to either chemically or green synthesized AgNPs; however, they showed a slight increase in resistance to ionic silver. Genomics analysis identified polymorphism in genes in a green synthesized AgNP-resistant line including but not limited to the multidrug efflux transporter system (*EmrAB*), DUF4756 family protein (*D1792_RS05680*), putative zinc-binding protein YnfU/cold shock-like protein (*ynfU/cspB*) and imcF-related family protein (*D1792_RS10035*). Bacterial resistance to chemical AgNPs involves specific polymorphisms in key bacterial components like the RNA polymerase sigma factor (*RpoE*) and the EmrAB efflux pump. Collectively, the method used to synthesize the AgNPs influences their antibacterial efficacy and the likelihood of bacteria developing resistance. Understanding this interaction is vital for developing effective and resistance-controlled applications of AgNPs across medicine, environmental science, and industry.

## 1. Introduction

Multidrug-resistant bacteria present a significant public health crisis because they are increasingly resistant to antibiotics, leading to limited treatment choices, poorer patient outcomes, increased spread of infection, and a greater burden on healthcare systems [1,2]. These resistant organisms threaten the effectiveness of antibiotics, making common infections difficult or impossible to treat and potentially leading to more deaths and higher healthcare costs [3,4]. Given the rise in patient mortality from antimicrobial resistance, researchers are re-evaluating the use of metals like silver as antimicrobials to combat resistant bacteria.

Historically, silver held dual significance: it was seen as a mystical substance in folklore while also being valued for its hygienic and therapeutic properties. Ancient cultures, such as the Greek and Roman civilizations, applied it to the skin via lotions and ointments to treat various ailments [5]. Moreover, long before the scientific understanding of microbes emerged, silver’s antimicrobial capabilities were utilized to purify liquids, such as water [6]. Silver acts as a multifaceted antimicrobial agent both as ions and nanoparticles that destroys cell walls, disrupts respiration and reproduction, and triggers toxic reactive oxygen species, offering a promising solution to combat the escalating crisis of antimicrobial resistance [7,8]. Silver nanoparticles (AgNPs) effectively combat multidrug-resistant bacteria by disrupting cellular functions and defenses through a complex combination of mechanisms, including ROS production, that depend on the specific traits of both the particles and the targeted microbes [9,10,11,12,13,14,15,16].

The production of nanoparticles can be accomplished using a range of methods, generally categorized into chemical and green synthesis [17]. While conventional chemical synthesis often relies on harsh chemicals and produces toxic waste, green synthesis offers a more environmentally friendly alternative by utilizing biological resources like plants, mushrooms and microorganisms [18]. Green synthesis offers a safer, eco-friendly alternative to conventional chemical methods. By utilizing renewable natural extracts and ambient conditions, it eliminates toxic solvents and hazardous catalysts. This approach yields harmless byproducts (like water and nitrogen) and natural biomolecular coatings, resulting in highly biocompatible materials for pharmaceutical and biomedical uses.

During the green synthesis of silver nanoparticles, biological organisms (like plants and microbes) supply naturally occurring bioactive molecules. These compounds act as powerful, eco-friendly reducing and stabilizing agents that transform dissolved metal ions into solid nanoparticles [11]. Additionally, proteins and other biomolecules serve as capping/stabilizing agents, preventing nanoparticle agglomeration and ensuring stability. This process creates biocompatible nanoparticles with improved antimicrobial and antioxidant properties, offering a safe, cost-effective, and sustainable approach to nanotechnology, and thus minimizes the use of harmful chemicals and reduces the generation of toxic byproducts, making it a more sustainable option [19].

Silver nanoparticles (AgNPs), one of the most common types of nanoparticles, are synthesized using both traditional chemical techniques and sustainable green synthesis approaches. Regardless of how they are synthesized, AgNPs attack microbes in two main ways. First, they attach to the cell surface, causing structural damage like gaps, destabilization, and leakage of the cell’s contents. Second, they release ions that attack the cell from within, disrupting its internal machinery and causing lethal oxidative stress [11,20,21,22]. While the antimicrobial effectiveness of both chemically synthesized and green synthesized AgNPs has been established, there is a gap in comparative research directly assessing which method yields more potent antimicrobial activity, though some studies suggest green synthesis is more effective and safer due to its eco-friendly nature and reduced toxicity [23,24]. Research comparing the effectiveness of chemically synthesized and green synthesized AgNPs has been identified as an area requiring further investigation. Considering the projected use of AgNPs for bacterial control, a comparative study is critical to assess the potential for bacterial resistance to evolve against nanoparticles produced through chemical and green synthesis.

Repeated exposure to sub-lethal doses of AgNPs can lead to the development of resistance in bacteria, particularly Gram-negative strains, for example, *Escherichia coli* and *Pseudomonas aeruginosa* [25,26]. This resistance stems from two primary mechanisms. The first is phenotypic changes, where bacteria alter their characteristics or behavior without genomic modifications. For example, in *E. coli*, resistance arises through the production of flagellin, a protein that aggregates the AgNPs and reduces their antibacterial activity [27]. Phenotypic silver resistance in bacteria can also occur through the formation of extracellular polymeric substances for silver sequestration or via modifications to outer membrane proteins to restrict silver uptake [28]. Additional resistance mechanisms include the expression of efflux pumps for active silver removal and the use of silver-binding proteins to sequester ions [29]. The former can sometimes involve underlying genomics alterations. Unlike genomic resistance, which involves heritable changes, phenotypic adaptations might be reversible when the nanoparticles are no longer present.

Regarding genomic mutations, bacteria can acquire heritable resistance through mutations in their genes. One example is the *CusS* mutation in *E. coli*, which affects the efflux pump responsible for expelling silver ions, contributing to resistance to AgNPs [25]. While both chemically synthesized AgNP and green synthesized AgNP methods are employed for AgNP production, there is a recognized need for more extensive studies that directly compare their properties and effectiveness, particularly in specific applications like antimicrobial activity. This study assessed whether bacteria could acquire resistance to AgNPs produced via chemical or green synthesis routes. Studying bacterial resistance to different silver AgNP formulations is vital for addressing the antibiotic resistance crisis. This research offers valuable insights into resistance mechanisms, facilitates comparison between synthesis methods, and aids in developing sustainable antimicrobial strategies. In this study, chemically synthesized AgNPs were acquired as a dispersion from Sigma-Aldrich, St. Louis, MO, USA, while green synthesized AgNPs were derived from reishi *Ganoderma lucidum* mushrooms. Using *G. lucidum* for the green synthesis of AgNPs is a safe, cost-effective, and environmentally friendly method. The mushroom’s rich bioactive compounds act as natural reducing and stabilizing agents, eliminating the need for toxic chemicals. Furthermore, *G. lucidum* is abundant in the Winston-Salem and Appalachian regions of North Carolina.

## 2. Materials and Methods

### 2.1. Materials

The materials utilized were silver nanoparticles (catalog number 730793; Sigma-Aldrich, St. Louis, MO, USA), Bel Art™ Lab Companion Cabinet Style Vacuum Desiccator (catalog number 08-648-113; Fisher Scientific, Hampton, NH, USA), 96-well plates (catalog number 269787; Thermo Scientific™, Hampton, NH, USA), silver chloride (6.0–50.0 mg/L) (99.9%; catalog number 011421.14; Thermo Scientific, Hampton, NH, USA), 50 mL Erlenmeyer flasks (catalog number 4117-0125; Thermo Scientific™, Hampton, NH, USA), nutrient broth (Thermo Scientific™, Hampton, NH, USA), the GloMax^®^-Multi Microplate (catalog number TM297; Promega, Madison, WI, USA) Ethanol, Absolute 200 Proof (catalog number BP28184; Fisher Scientific, Hampton, NH, USA), an Eisco™ Erlenmeyer Flask conical flask (catalog number S15470; Fisher Scientific, NH, USA), 0.5 McFarland standard (catalog number R20410; Fisher Scientific, Hampton, NH, USA), amber bottles (catalog number FB02911900; Fisher Scientific, Hampton, NH, USA) and a Corning^®^ bottle-top vacuum filter system (catalog number 431155; Corning, NY, USA).

### 2.2. Bacterial Strains and Experimental Growth Conditions

The antibacterial effectiveness of AgNPs, created through both green and chemical synthesis methods, was assessed against Gram-negative *Escherichia coli* ATCC# 25922 obtained from the Department of Biological Sciences at Winston Salem State University in Winston Salem, NC, USA. The bacterial strain under investigation was cultured overnight in nutrient broth (Fisher Scientific, NH, USA) at 37 °C with orbital shaking at 160 rpm. For the tested strain, the inoculum size was adjusted to a concentration of 1.5 × 10^8^ CFU/mL, referencing a 0.5 McFarland standard (Fisher Scientific, Hampton, NH, USA).

### 2.3. Extract Preparation and Synthesis of Silver Nanoparticles

Freshly harvested *Ganoderma lucidum* (also known as reishi) mushrooms were dehydrated (Thermo Scientific™, Hampton, NH, USA) without heat for three days, pulverized to ≤2 mm particulates, and double-bagged. A 100 g portion of this powder was extracted in 0.5 L of distilled water in a conical flask (Fisher Scientific, NH, USA) at 50 °C, shaken at 150 rpm for 48 h, centrifuged at 3000 rpm for 10 min, and vacuum-filtered through a Corning^®^ bottle-top vacuum filter system (Corning, NY, USA) prior to storage at 4 °C. The green synthesis of AgNPs was carried out with reishi extract, adhering to the methodology detailed by Ewunkem et al. [11]. Briefly, 10 mL of reishi extract was combined with 1 mM AgNO_3_ (100 mL) in a conical flask and continuously agitated at room temperature. A yellowish-brown color confirmed the synthesis of AgNPs, and the mixture was maintained at a pH of 7. The color remained unchanged even after 168 h, suggesting the green synthesized AgNPs were dispersed in the solution. The chemically synthesized AgNPs used in this study were purchased from Sigma-Aldrich (St. Louis, MO, USA; catalog number 730793). During their preparation, a controlled chemical reduction process of a silver salt was utilized. In this process, a reducing agent converts silver ions into elemental silver, while sodium citrate acts as a capping and stabilizing agent to regulate particle growth and prevent aggregation.

### 2.4. Minimum Inhibitory Concentration (MIC) of Green Synthesized and Chemically Synthesized Silver Nanoparticles

The minimum inhibitory concentration (MIC) of green synthesized and chemically synthesized AgNPs was determined by serially diluting them in nutrient broth within 96-well plates. Five selected colonies were grown overnight to confluency in 10 mL of nutrient broth (NB). A subsequent MIC assay was performed on an aliquot of the culture to determine the sublethal concentration of AgNPs for selection purposes. The experimental setup involved diluting a bacterial overnight culture to an OD 600 nm of 0.05 and plating it. The effects of green synthesized AgNPs (0–4.5 µM) and chemically synthesized AgNPs (0–8.0 mg/L) were then assessed across a range of concentrations. All experiments were conducted in triplicate for accurate comparison and to ensure robustness and statistical reliability. Turbidity, as an indicator of bacterial growth in NB, was quantified by measuring the optical density at 600 nm using a GloMax^®^-Multi Microplate reader and clear polyester 98-well plates [21,22]. Measurements were acquired at both 0 and 24 h, and the initial 0 h values were subtracted from the 24 h values for statistical evaluation. The sublethal concentrations of AgNPs for selection were established using an MIC assay with the ancestral *E. coli* strain [25]. The assay found a sublethal concentration of 0.8 mg/L for the chemically synthesized AgNPs (Sigma-Aldrich, St. Louis, MO, USA) and 0.02 mM for the green synthesized AgNPs from reishi mushroom.

### 2.5. Evolutionary Experiments

Stock cultures were maintained through daily 1:100 serial dilutions in fresh nutrient broth (NB) for seven days, as described by Graves et al. [25]. This propagation aimed to ensure adequate regrowth of the bacteria before being subjected to selection with green synthesized and chemically synthesized AgNPs. For comparative purposes, control samples were established by taking five separate 0.1 mL aliquots of the stock culture and diluting them 1:100 in NB. The bacterial cultures were grown for 24 h in 50 mL Erlenmeyer flasks and placed in a shaking incubator at 115 rpm and 37 °C. Each replicate was initiated from a unique colony on an agar plate isolated via serial dilution from the ancestral stock culture. These samples were placed in separate flasks and allowed to grow for 24 h before being transferred to fresh media. Fifteen bacterial strains, initially grown as individual colonies, were cultured in sterile NB within 50 mL Erlenmeyer flasks. These strains were then divided into three groups of five flasks each: a control group (C1–C5) receiving only *E. coli* in NB, a treatment group (MN1–MN5) exposed to green synthesized AgNPs, and another treatment group (CN1–CN5) exposed to chemically synthesized AgNPs. All cultures, including the treatment groups, were maintained through daily subculturing. This involved transferring 0.1 mL of an existing culture into 9.9 mL of fresh, sterile NB each day. Each daily subculturing cycle led to a significant increase in population density, growing from approximately 10^7^ to 10^9^ cells per mL. Repeating the selection experiments multiple times is essential to determine if adaptations arise consistently in response to the same selective pressures. This repetition, known as replication, helps rule out chance events and build confidence in the findings.

### 2.6. Preparation Techniques for Scanning Electron Microscopy

After 21 days of selection, nanoparticle-resistant bacteria, alongside control and ancestral cell lines, were prepared for morphological analysis using a Carl Zeiss Auriga-BU FIB FESEM (Carl Zeiss, Oberkochen, Germany) [11,21,22]. The preparation process involved centrifuging the bacterial suspensions, followed by resuspending the resulting pellets in PBS. The cells were then fixed using glutaraldehyde before being dehydrated through a graded series of ethanol solutions in preparation for scanning electron microscopy (SEM) analysis.

### 2.7. Characterization of Synthesized Silver Nanoparticles

The chemical and green synthesized AgNPs were characterized according to the method described by Ewunkem et al. [11]. UV-vis spectra were measured using a GENESYSTM 180 UV-Vis Spectrophotometer (Fisher Scientific, USA) within the range of 200–1000 nm. Ambient temperature FTIR spectra were acquired using a Thermo Fisher Nicolet iS50 spectrometer (Thermo Fisher Scientific Inc. Portsmouth, NH, USA) over a range of 400–4000 cm^−1^, with a resolution of 4 cm^−1^ and an average of 32 scans. Additionally, dynamic light scattering and zeta potential analyses were performed at 25 °C on green synthesized reishi AgNP samples using a Malvern Zetasizer Ultra (Malvern Panalytical Ltd., Malvern, UK). All 0.7 mL samples were analyzed in triplicate to ensure reproducibility. The manufacturer supplied the absorbance data for the chemically synthesized silver nanoparticles (https://www.sigmaaldrich.com/US/en Accessed on 24 April 2026). The shape and size of AgNPs were determined by the JEOL JEM-2100 plus TEM, a multipurpose, 200 kV analytical electron microscope with an ultrahigh TEM resolution as high as 0.19 nm (in UHR configuration) (JEOL USA, Inc. Peabody, MA, USA) at the Joint School of Nanoscience and Nanoengieering (JSNN), Greensboro, NC, USA. The nanoparticles were drop-coated in a copper grid with a size 300 mesh and were observed at 300 kV.

### 2.8. Analysis of Phenotypic Growth Assays over a 24-Hour Period

Bacterial resistance to AgNPs can potentially lead to cross-resistance although the specific mechanisms can differ [30]. At 21 days of evolution (day 21 was chosen because it was the earliest time point at which nanoparticle resistance was observed), phenotypic assays were conducted to assess potential pleiotropic effects associated with nanoparticle adaptation to ionic silver. Furthermore, the green synthesized AgNP-selected populations, along with the control populations, were assessed for fitness and adaptability in increasing concentrations of chemically synthesized AgNPs vice versa. Also, the fitness and cross-adaptability of selected populations of chemically synthesized AgNP populations were assessed by measuring their response to escalating levels of ionic silver and green synthesized AgNPs. These values were compared with the five samples of the *E. coli* ancestor, which grew overnight in NB. The range used was 0–5.4 mg/L for ionic silver, and 0–10 mg/L of green synthesized AgNPs and chemically synthesized AgNPs. Turbidity (optical density at 650 nm) was measured to quantify bacterial growth at 0 and 24 h using a GloMax^®^-Multi Microplate Reader and clear polyester 98-well plates. The 0 h readings were subtracted from the 24 h readings for statistical analysis to determine the change in turbidity over time.

### 2.9. Whole-Genome Sequencing

After 21 days of selection, bacterial genomic DNA was isolated and sequenced at SeqCoast Genomics using the methods described by Ewunkem et al. [22]. This was carried out for both green synthesized and chemically synthesized AgNPs. DNA was extracted from each population using the DNeasy 96 PowerSoil Pro QIAcube HT Kit (Qiagen, Redwood City, CA, USA) according to the manufacturer’s instructions. Subsequently, genomic libraries for sequencing were prepared using the Illumina DNA Prep tagmentation kit and IDT For Illumina Unique Dual Indexes (Illumina, San Diego, CA, USA) as per the manufacturer’s manuals. DRAGEN v4.2.7, which is integrated into the NextSeq 2000 system (Illumina, San Diego, CA, USA) was used to carry out demultiplexing, read trimming, and run analytics.

### 2.10. Statistical Analysis

To evaluate the effects of different silver treatments, a general linear model was fitted to the 24 h growth data (optical density) using SPSS (version 29). The analyzed groups included green synthesized AgNPs, chemically synthesized AgNPs, and ionic silver, all compared against controls. Mean differences were determined using Bonferroni’s multiple comparisons test, and plots were generated using GraphPad Prism (version 10).

## 3. Results

### 3.1. Characterization of Green Synthesized and Chemically Synthesized Silver Nanoparticles

**Figure 1:**
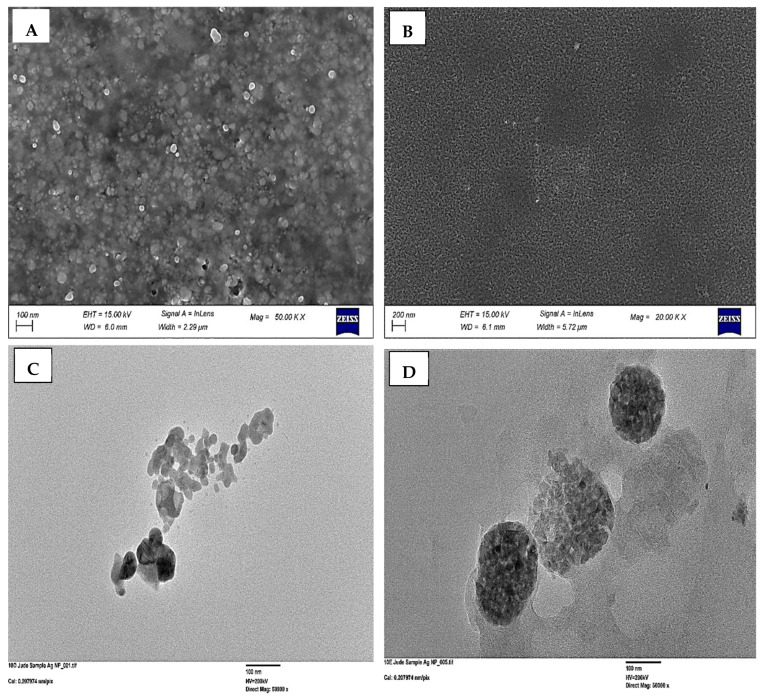
Morphological characterization of silver nanoparticles (AgNPs) produced by green synthesis (using reishi mushroom) and chemical synthesis, as visualized by electron microscopy. (**A**) SEM of green synthesized AgNPs, (**B**) SEM of chemically synthesized AgNPs, (**C**) TEM of green synthesized AgNPs, and (**D**) TEM of chemically synthesized AgNPs.

**Figure 2:**
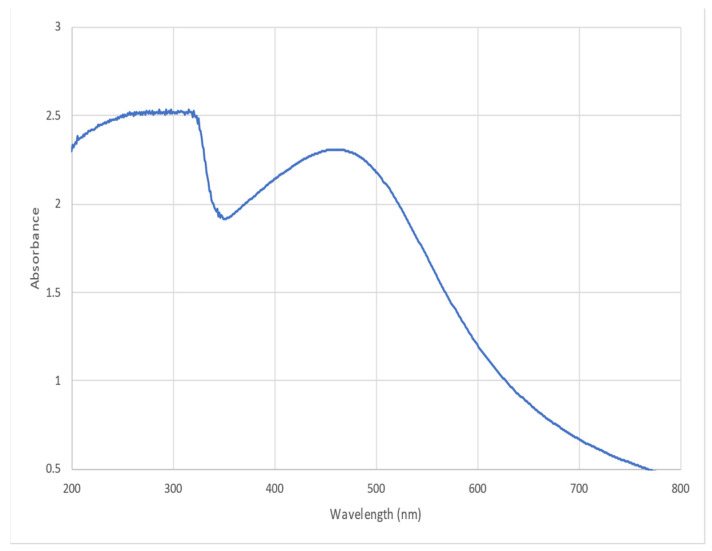
The ultraviolet–visible (UV-Vis) spectra of AgNPs created through green synthesis.

The electron microscopic observations confirmed the formation of the AgNPs in the two samples analyzed (Figure 1). The results obtained from SEM and TEM provided detailed information on the dispersity, size distribution, and morphology of the green synthesized and chemically synthesized AgNPs. The green synthesized AgNPs were spherical or near spherical, and several ones were irregular (Figure 1A,C). The analysis of data obtained from SEM and TEM micrographs of the chemically synthesized AgNPs confirmed the formation of spherical nanoparticles with differing sizes (Figure 1B,D). The spectrophotometric data independently corroborated the findings from both the SEM and TEM imaging, and revealed that both the green synthesized and the chemically synthesized AgNPs exhibited a pronounced and characteristic optical signature. Specifically, the green synthesized AgNPs displayed a very strong absorption peak in the visible light spectrum, centering around the 440–450 nanometer (nm) range (Figure 2). The chemically synthesized AgNPs displayed a comparable peak. This high absorption peak is a critical and well-understood phenomenon known as the surface plasmon resonance (SPR). Zeta potential analysis of the green synthesized AgNPs revealed a surface charge of −25.76 mV at room temperature, denoting excellent colloidal stability at a neutral pH (Supplementary Figure S1). Additionally, FTIR analysis (Supplementary Figures S2 and S3) identified the functional groups responsible for capping the AgNPs, with sharp transmittance peaks appearing at 1650.04 cm^−1^ and 3400.00 cm^−1^ in the extract.

### 3.2. E. coli Resistance to Green Synthesized Silver Nanoparticles

**Figure 3:**
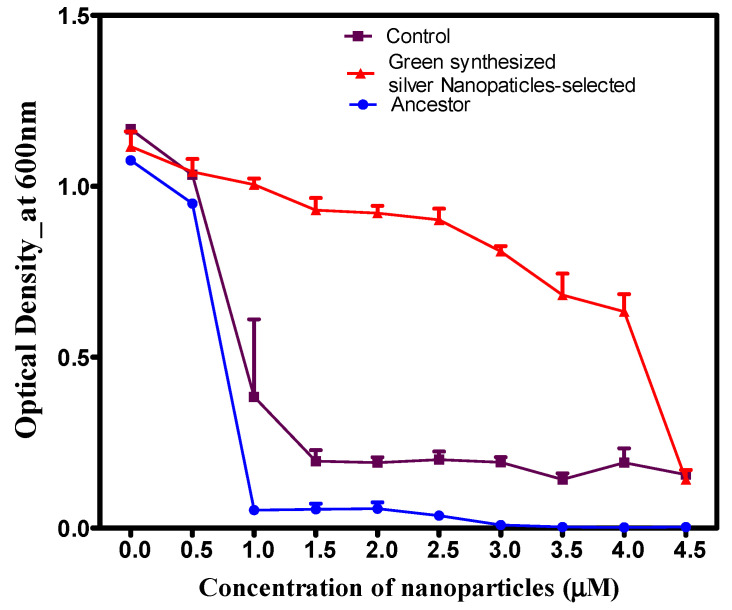
Average 24 h growth of *E. coli* populations evolved for 21 days in the presence of increasing concentrations of green synthesized AgNPs. In a 21-day experiment, green synthesized-derived populations of *E. coli* were grown in nutrient broth containing 0.02 mM of silver nanoparticles, while control populations were grown in plain nutrient broth. Ancestral *E. coli* populations were grown in nutrient broth for 24 h as a baseline. The green synthesized-derived populations demonstrated significantly higher growth than both the control and ancestral populations.

After 21 days of selective pressure, all populations showed reduced growth as the concentration of mushroom-synthesized silver nanoparticles increased (Figure 3). Optical density (OD) was significantly affected by both population and concentration (*p* < 0.001). Significant differences in OD were observed across all three populations (ancestor, control, and green synthesized AgNPs), with highly significant differences (*p* < 0.001) in all pairwise comparisons. However, the population specifically selected with these AgNPs demonstrated significantly (*p* < 0.001) better growth across all tested concentrations compared to the control and ancestral populations. This green synthesized AgNP-resistant population exhibited substantially increased growth (with greater optical densities, indicating more cells) at concentrations ranging from 0.5 to 4.0 µM, and this improvement was statistically significant (*p* < 0.001) compared to the ancestral and control groups.

### 3.3. Exposure to Mushroom-Derived Nanoparticles Promotes Resistance to Ionic Silver

**Figure 4:**
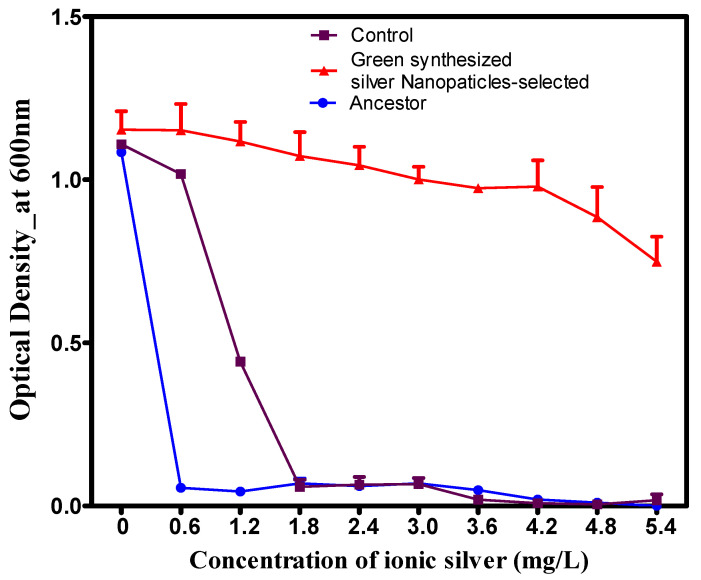
Growth of *E. coli* populations after 21 days of evolution in gradually increasing ionic silver concentrations: the growth of green synthesized-derived AgNPs populations was notably higher than that observed in either the control or ancestral populations.

The same mechanisms that allow bacteria to develop resistance to green synthesized AgNPs can also protect them against ionic silver [31]. When exposed to increasing concentrations of ionic silver (0.0–5.4 mg/L), populations previously exposed to green synthesized AgNPs demonstrated a significantly (*p* < 0.001) faster and more robust growth after 24 h compared to both control groups and the original, unexposed ancestor population, as shown in Figure 4. Concentration, population size, and their interaction all had highly significant effects on OD (*p* < 0.001). Furthermore, post hoc analysis revealed that the ancestor, control, and green synthesized population were all significantly different from each other (*p* < 0.001).

### 3.4. Cross-Resistance Between Green Synthesized and Chemically Synthesized Silver Nanoparticles

**Figure 5:**
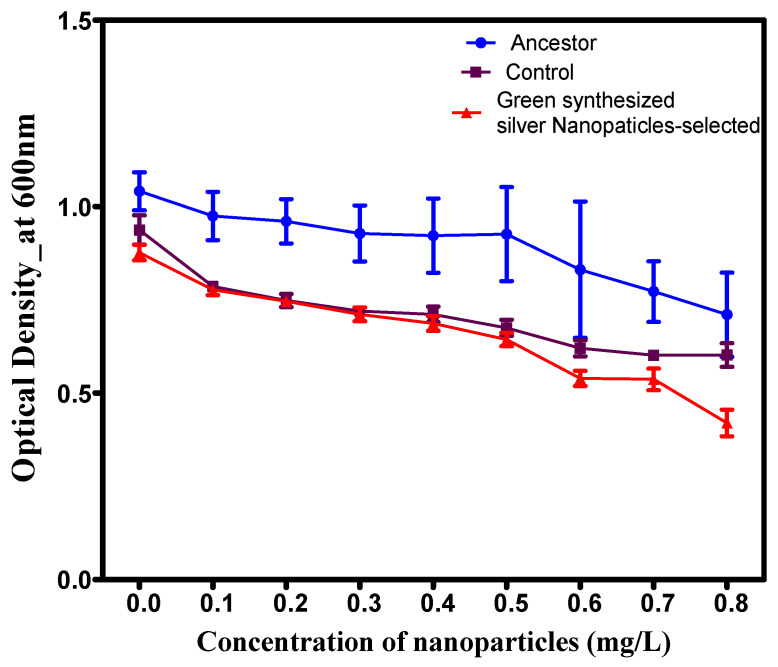
Growth of *E. coli* populations after 21 days of evolution in increasing concentrations of chemically synthesized AgNPs. The growth of nanoparticle populations synthesized using mushrooms was significantly lower than that observed in both ancestral and control populations.

After 21 days of selection, the green synthesized AgNP populations displayed a significantly (*p* < 0.05) inferior growth relative to the controls and ancestral strain across all concentrations of chemically synthesized AgNPs (Figure 5). There were significant main effects of both the population (*p* < 0.001) and concentration (*p* < 0.001) on OD. However, their interaction was not significant (*p* = 0.223), suggesting that these factors acted independently. Pairwise comparisons showed significant OD differences across all populations (*p* < 0.05). The ancestor group had the highest OD, surpassing both the control (*p* < 0.001) and chemical nanoparticles (*p* < 0.001). This difference was extremely pronounced, with increasing optical densities found at lower concentrations (0.0–0.8 mg/L), while higher concentrations showed steady growth, attributable to a more constant outcome of optical densities. Finally, the control group yielded a significantly higher OD than the chemical nanoparticles (*p* = 0.004).

### 3.5. E. coli Response to Chemically Synthesized Silver Nanoparticles

**Figure 6:**
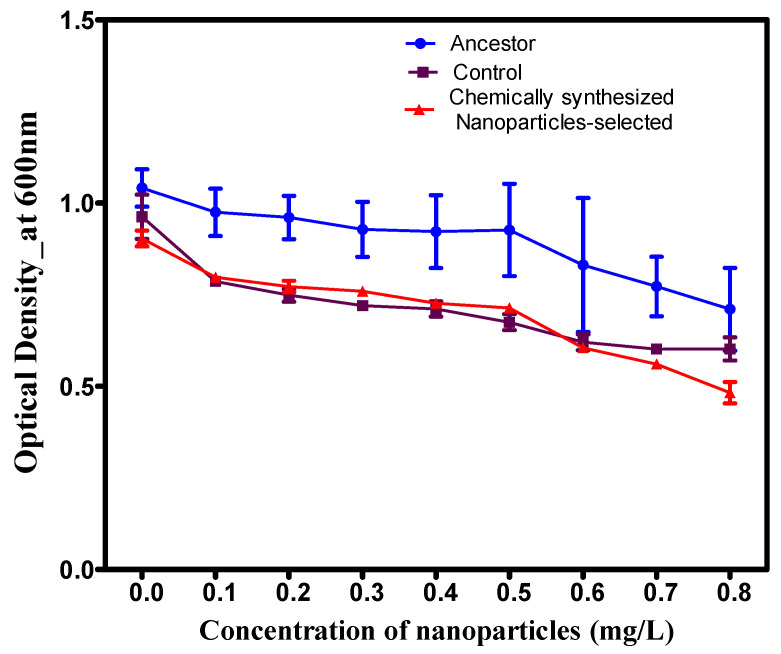
Average 24 h growth of *E. coli* populations evolved for 21 days in the presence of increasing concentrations of chemically synthesized AgNPs. In a 21-day experiment, chemically synthesized AgNP populations of *E. coli* were grown in nutrient broth containing 0.5 mg/L of AgNPs, while control populations were grown in plain nutrient broth. Ancestral *E. coli* populations were grown in nutrient broth for 24 h as a baseline. The population growth rate of ancestral populations was considerably higher than that of the control and selected populations.

After 21 days of exposure to increasing concentrations of chemically synthesized AgNPs (from 0 to 8.0 mg/L), a dose-dependent reduction in growth was observed across all populations tested (Figure 6). Notably, ancestral populations exhibited significantly enhanced growth compared to both selected and control populations, with a statistical significance of *p* < 0.0001. However, the selected and control populations showed no significant difference in growth at AgNP concentrations ranging from 0 to 7.0 mg/L. It was only at higher concentrations that the selected population demonstrated a significantly reduced growth compared to the control populations. Furthermore, there were significant effects for both concentration (*p* < 0.001) and population (*p* < 0.001) on OD, indicating that these factors independently affect OD levels. However, the concentration–population interaction was not significant (*p* = 0.376). Pairwise comparisons showed significant differences between the ancestral population and the other two groups, while no significant differences were found between the control and chemically synthesized silver nanoparticle populations (*p* > 0.001).

### 3.6. Exposure to Chemically Synthesized Silver Nanoparticles Promotes Resistance to Ionic Silver

**Figure 7:**
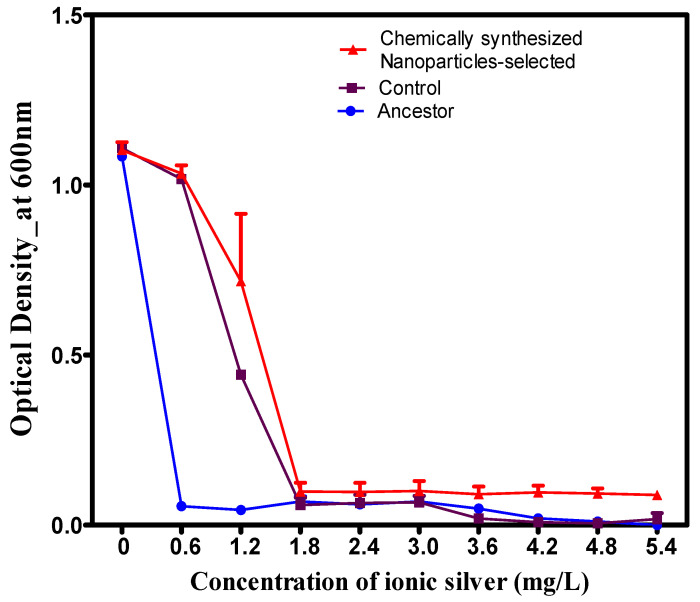
Growth of *E. coli* populations after 21 days of evolution in gradually increasing ionic silver concentrations: The *E. coli* population that underwent selection in a chemically synthesized AgNPs exhibited significantly (*p* < 0.05) higher growth compared to both the control and ancestral populations.

Resistance mechanisms for silver nanoparticles and ionic silver often overlap or influence each other, so resistance to one can confer resistance to the other, but not always perfectly. When exposed to increasing concentrations of ionic silver (0.0–5.4 mg/L), populations previously exposed to chemically synthesized AgNPs demonstrated significantly (*p* < 0.001) faster, enhanced, and more robust growth after 24 h compared to both control groups and the original, unexposed ancestral population, as shown in Figure 7. All factors and their interactions significantly influenced OD (*p* < 0.001). The concentration and population both showed strong main effects, and a significant interaction effect indicated that concentration impacts OD differently depending on the population. Pairwise comparisons revealed significant differences across all three groups (*p* < 0.001).

### 3.7. Cross-Resistance Between Chemically and Green Synthesized Silver Nanoparticles

**Figure 8:**
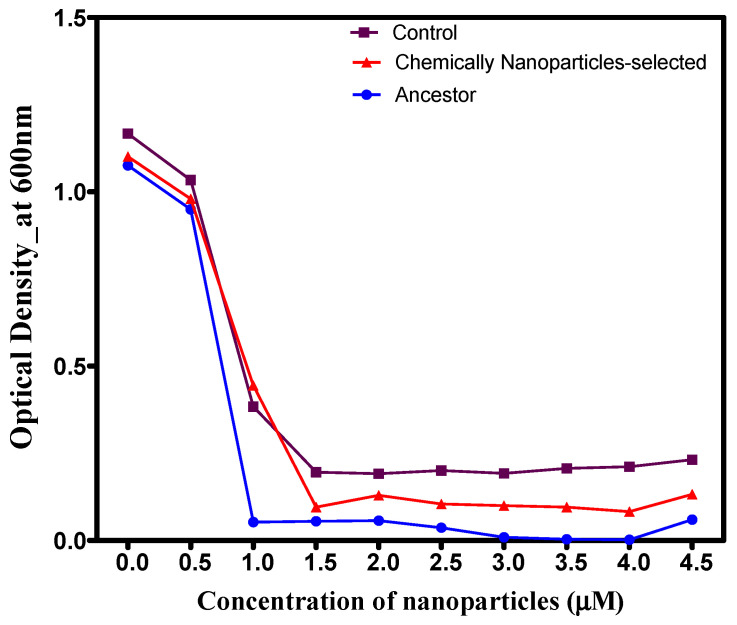
Growth of *E. coli* populations after 21 days of evolution in increasing concentrations of green synthesized AgNPs. *E. coli* populations exposed to chemically synthesized AgNPs exhibited significantly reduced growth compared to the control group but grew significantly faster than the ancestral population.

After 21 days of selection, *E. coli* evolved in the presence of chemically synthesized AgNPs exhibited significantly (*p* < 0.001) improved growth compared to the ancestral population when exposed to increasing concentrations (0.5 to 4.5 µM) of green synthesized AgNPs (Figure 8). However, the same selected *E. coli* population showed reduced growth compared to the control population. Both population and concentration significantly affected optical density (*p* < 0.001), with no significant interaction (*p* = 0.054). Pairwise comparisons revealed significant differences between all groups (*p* < 0.001).

### 3.8. Genomic Analysis

Whole-genome resequencing was conducted on all populations after 21 days of selection to identify polymorphisms (indels and single-nucleotide polymorphisms) related to the selection regime. Comparison of these sequences with the *Escherichia coli* strain ATCC 25922 reference genome (NCBI: NZ_CP032088.1) enabled the detection of selection-associated genetic variations. Previously documented ancestral mutations are described by Ewunkem et al. [32].

Within 21 days of continuous exposure to green synthesized AgNPs, the bacterial population (MNP1-MNP5) underwent significant adaptive changes. This evolutionary response was characterized by selective sweeps, a process where beneficial mutations rapidly spread throughout the population, effectively “sweeping” away genetic variation in adjacent DNA regions. This rapid adaptation was genetically complex, driven by mutations occurring in a total of 41 distinct genes, including key genes such as *emrR*, *diaA*, *relA*, *D1792_RS05680*, *D1792_RS06990/D1792_RS26615*, *uacT*, *pstC/pstS*, *glnH*, *ais/arnB*, *fiu*, *ydjF*, *iroE*, *sirB2*, *ynfU/cspB*, *baeS*, *D1792_RS10035*, *pdeN*, *lolE*, *dgoK*, *xapA*, *lysO/aqpZ*, *yfhb*, *D1792_RS02575*, *D1792_RS24525*, *sstT*, *cysB/ymiA*, *D1792_RS14205*, *D1792_RS20645/D1792_RS20650*, *rplJ*, *mdtE*, *cyoA*, *cysJ/queD*, *sirB2*, *D1792_RS02005*, *D1792_RS02575*, *pgaC*, *gpJ*, *D1792_RS05680*, *rplC*, *fdrA*, *fryC*, *D1792_RS25790* and *D1792_RS06050*. Polymorphisms in the *glnH* gene were universally observed in all the populations studied. Interestingly, among the various mutations documented, a single specific mutation was found to be particularly prevalent, with its frequency surpassing 0.5. The frequency and genomic distribution of adaptive mutations are cataloged in Table 1, and the associated genes are described in Table 2.

Analysis of the chemically synthesized AgNP-selected populations (CNP1-CNP5) revealed the presence of polymorphisms and evidence of selective sweeps, presented in Table 3 along with their corresponding frequencies and genomic locations. Among these, 48 polymorphisms in the CNP-selected populations demonstrated a notable increase in frequency, from 0.0 to 0.58, within the specified genes: *diaA*, *glnH*, *nadA*, *uacT*, *cyoA*, *yfhb*, *ycdZ*, *gpJ*, *mdoG*, *rplJ*, *D1792_RS20520*, *nrdH*, *sucD*, *sirB2*, *baeS*, *atpG*, *emrR*, *D1792_RS02005*, *ais/arnB*, *lysO/aqpZ*, *relA*, *ybhR*, *rplC*, *D1792_RS25790*, *D1792_RS02575*, *cysB/ymiA*, *rpoE*, *D1792_RS03435*, *rcsC*, *xapA*, *D1792_RS20645/D1792_RS20650*, *ydjF*, *nadA*, *prpR/prpB*, *D1792_RS17175*, *pqiB*, *nrfB*, *D1792_RS15055*, *fiu*, *mgtT/dgcZ*, *fdrA*, *D1792_RS05680*, *gltI/D1792_RS22555*, *irp1*, *D1792_RS06990/D1792_RS26615*, *thiB/sgrR*, *pgaC* and *D1792_RS11095.* Polymorphisms in *diaA* and *cyoA* were universally present across all populations. Table 2 describes the genes in which these mutations are found.

Table 4 illustrates the polymorphism and mutation frequencies within control populations (C1–C5) at day 21. Notably, the control populations collectively possessed 38 putative polymorphisms, with two reaching a frequency above 0.5. Specifically, these polymorphisms occurred in *lysO/aqpZ*, *ais/arnB*, *cysB/ymiA*, *relA*, *ulaG D1792_RS03435*, *glnH*, *uacT*, *D1792_RS01330*, *bfd/chiA*, *rplJ*, *D1792_RS20520*, *prpR/prpB*, *diaA*, *yjcE/D1792_RS17315*, *nadA*, *mdoG*, *pstC/pstS*, *D1792_RS24525*, *D1792_RS02575*, *xapA*, *rhaD*, *gnsB*, *fryC*, *ydjF*, *cyoA*, *pgaC*, *baeS*, *sstT*, *rnb*, *gpJ*, *D1792_RS20645/D1792_RS20650*, *nrfB*, *D1792_RS02005*, *D1792_RS06050*, *D1792_RS11890*, *fdrA* and *pic/D1792_RS20755.* All populations consistently displayed polymorphisms in *ais/arnB*, *glnH* and *diaA*.

Adaptation and morphological changes in *E. coli* a under selective pressure of green synthesized and chemically synthesized silver nanoparticles.

**Figure 9:**
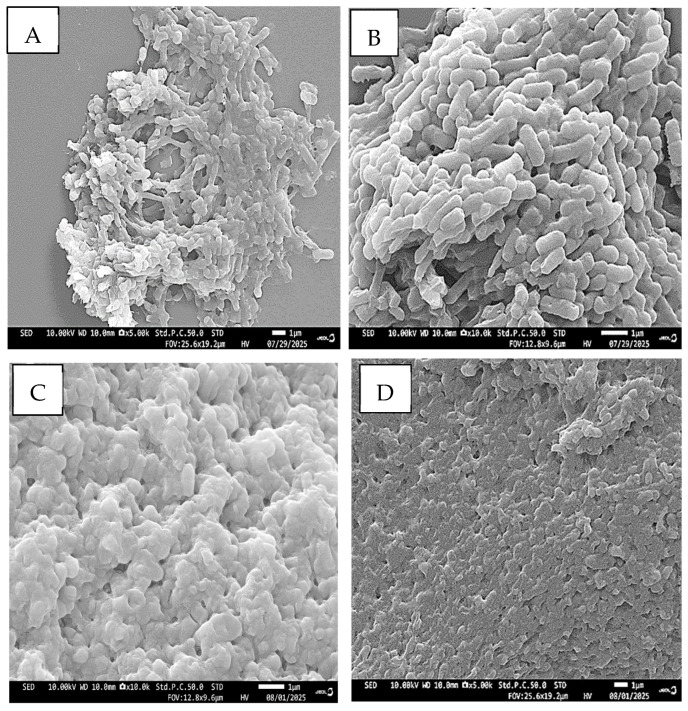
SEM images of control bacterial cells and cells treated with green synthesized AgNPs and chemically synthesized AgNPs after 24 h. (**A**,**B**) Control *E. coli*; (**C**) treated with green synthesized AgNPs; (**D**) *E. coli* treated with chemically synthesized AgNPs. Untreated cells appeared smooth. The nanoparticles induced fragmentation and irregularities in the bacterial envelope. Additionally, AgNPs adsorbed onto the bacterial surface, forming aggregates of varying sizes.

Based on scanning electron microscopy (SEM) analysis conducted after 21 days of selection (Figure 9), *E. coli* cells treated with green synthesized and chemically synthesized AgNPs exhibited distinct morphological changes when compared to control and ancestral populations. Green synthesized AgNPs (from mushroom extract) at 0.02 mM concentration resulted in longer cells, measuring approximately 1.9 µm in length. Furthermore, surface abnormalities like voids, pits, and depressions were observed. Cells selected with chemically synthesized AgNPs (at 0.8 mg/L) were notably shorter than the green synthesized nanoparticle-treated cells, averaging around 1.5 µm in length. Control and ancestral cells appeared significantly shorter, with average lengths of 0.35 µm and 0.31 µm, respectively. No statistically significant difference in length was noted between these two groups.

## 4. Discussion

The escalating problem of antibiotic resistance necessitates the exploration of alternative treatments for bacterial infections, such as those employing silver and nanotechnology [33,34]. Among the alternatives, AgNPs show significant promise due to their high surface area, which enhances interactions with bacteria, and their ability to release silver ions, both contributing to their strong antibacterial properties [35]. Recent advancements in nanotechnology have focused on developing environmentally friendly methods for synthesizing AgNPs, known as green synthesis, in addition to conventional chemical synthesis methods [36,37]. This research aimed to understand the mechanisms of resistance development in *E. coli* by investigating the genomics and phenotypic changes that occur in the bacteria upon exposure to both green synthesized and chemically synthesized AgNPs. Furthermore, this research sought to elucidate the mechanisms by which selection pressures from green synthesized and chemically produced AgNPs confer resistance to ionic silver. Studying how bacteria resist differently synthesized AgNPs and how this relates to ionic silver is crucial for preventing the spread of multidrug-resistant pathogens and designing targeted nano-therapeutics. Additionally, this study provides insights into the mechanisms of adaptation and resistance to green synthesized and chemically synthesized AgNPs, highlighting both shared and distinct mechanisms of resistance.

This study details the green synthesis of silver nanoparticles using reishi mushroom extract as a reducing and stabilizing agent. The characteristics of these green synthesized AgNPs were then compared to those of conventionally produced AgNPs using spectrophotometry, SEM, and TEM. AgNPs were successfully synthesized using reishi mushroom extract indicated by a color change in the reaction mixture, which shifted from yellow-brown to reddish-brown within 24 h. A color change during nanoparticle synthesis indicates successful formation due to the surface plasmon resonance (SPR) phenomenon [38]. SPR is the collective vibration of electrons on a nanoparticle’s surface when they interact with light, causing the solution to absorb specific wavelengths of light and appear colored, often from yellow to brown for AgNPs [39]. This visual change serves as a simple, non-invasive initial indicator before further spectroscopic confirmation using UV-Vis spectroscopy. Consistent with previously published findings, UV-Vis spectroscopy was used to confirm the formation of AgNPs by identifying a characteristic SPR peak within the 400–500 nm wavelength range [40,41]. Under neutral conditions, green synthesized AgNPs demonstrate a zeta potential of −25.76 mV, confirming the creation of highly stable, negatively charged colloids. This significant negative charge induces interparticle repulsion, which inhibits aggregation and ensures excellent stability. Additionally, FTIR analysis confirms that biomolecules—specifically proteins, phenolic compounds, and terpenoids—serve as reducing and capping agents. The FTIR spectrum revealed multiple peaks, notably a broad band at 1650.04 cm^−1^ corresponding to the C=O (carbonyl) stretching, or Amide I band. This indicates the presence of proteins, enzymes, and flavonoids, which act as capping agents to prevent nanoparticle agglomeration. Additionally, a peak at 3400.00 cm^−1^ is assigned to O–H (hydroxyl) or N–H (amine) stretching, suggesting the involvement of alcohols, phenols, and amino acids. These functional groups, likely derived from mushroom extract biomolecules, are responsible for nanoparticle stabilization, a finding consistent with previous studies [39,40,41].

The morphology of the resulting nanoparticles was subsequently investigated through scanning electron microscopy (SEM) and transmission electron microscopy (TEM) [42]. Compared to chemical synthesis, which yields exclusively smaller, spherical AgNPs (10–30 nm), the green synthesis method produced larger nanoparticles in a wider variety of shapes, including cuboidal, triangular, hexagonal, and rod-like morphologies. Employing natural extracts for the green synthesis of AgNPs results in varied nanoscale architectures because of the chemical complexity of botanical extracts. The diverse phytochemicals act as reducing and capping agents during synthesis. For instance, reishi mushroom extracts contain a “natural cocktail” of biomolecules like beta [1,2,3] glucans, ganoderic acids, and triterpenoids [32,43]. These disparate components influence nanoparticle growth and stabilization differently, leading to a wide variety of shapes and sizes within a single batch [18].

Research, such as that presented by Güneş et al. [44], has highlighted that the precise physical and chemical characteristics of a reaction environment; specifically, the medium’s pH, the prevailing temperature, and the overall duration of the reaction are critical determinants of the resultant particle shapes or morphologies. This variability occurs because specific biomolecules within the system are designed to selectively adsorb onto crystal facets. By binding on these surfaces, these molecules effectively modulate the local growth rates of those specific faces, thereby steering the overall architectural development of the growing crystal into complex and distinct forms. The remarkable homogeneity in morphology and size of chemically synthesized AgNPs is a fascinating outcome of precisely controlled reaction kinetics, primarily governed by the principles of nucleation and growth in solution [45]. This high degree of uniformity, often referred to as monodispersity, is a defining characteristic of carefully optimized chemical methods, in stark contrast to the often-broader distributions seen in less controlled approaches like some biological syntheses, as previously explained [46]. The ability to achieve this control makes chemical synthesis a cornerstone of nanotechnology research and application, despite the use of potentially hazardous chemicals in some processes.

Differences in nanoparticle size and shape, which often vary between green and chemical synthesis, do affect bacterial resistance by influencing surface area, interaction with bacterial membranes, and the release of active components. Smaller nanoparticles, due to their higher surface-to-volume ratio, have better antimicrobial activity through enhanced contact with bacteria [47]. In contrast, differently shaped nanoparticles such as larger or irregular ones may trigger different bacterial responses, potentially contributing to bacterial resistance and suggesting a path for evolutionary adaptation. A correlation is anticipated between resistance to AgNPs and resistance to ionic silver. Irregularly shaped AgNPs often exhibit enhanced antibacterial activity compared to spherical ones, primarily due to their sharp edges more effectively damaging bacterial membranes and increasing the release of silver ions. These unique geometries lead to stronger bactericidal effects, which may slow the evolution of resistance. However, this enhanced activity is not a complete solution, as bacteria, particularly within protective biofilms, can still adapt and develop resistance mechanisms.

After 21 days of exposure to green synthesized AgNPs derived from reishi mushroom, the treated populations exhibited increased AgNP resistance compared to both the control and ancestral populations. In addition, resistance to green synthesized AgNPs correlated with increased resistance to ionic silver. Conversely, the green synthesized-selected populations showed inferior 24 h growth relative to the controls and ancestor populations in increasing concentrations of chemically synthesized AgNPs. Genomic analysis demonstrated that resistance alleles were already accumulating in the silver nanoparticle-resistant populations by day 21 of selection in green synthesized AgNPs derived from reishi mushroom. The increase in resistance to green synthesized AgNPs seems to be the result of major genomic and morphological changes. It is reported that bacterial resistance to antimicrobials stems from genomic changes (mutations, gene transfer) and phenotypic adaptations [48]. Genomics provides the blueprint (e.g., efflux pumps, target modification genes) while phenotypic changes (e.g., biofilms, metabolic shifts) create temporary, non-heritable resistance, often influenced by environmental factors and metabolism, highlighting a complex interplay between genes and the cell state in determining treatment failure [49].

Resistance to both green synthesized AgNPs and ionic silver in bacteria could have arisen from a specific genetic change: a selective sweep in the *emrR* gene. This mutation, found exclusively in bacterial populations exposed to green synthesized AgNPs, affects the function of the EmrAB multidrug efflux transporter system. The EmrAB efflux pump, regulated by the EmrR repressor, acts as a cellular defense mechanism [50,51]. In the presence of toxic compounds—for example, silver—these compounds bind to EmrR, triggering a conformational change that activates the EmrAB pump. This activation results in the expulsion of harmful substances from the cell, promoting the bacteria’s survival and adaptability to different environments and contributing to silver resistance, according to research by Lomovskaya et al. [50] and Xiong et al. [51].

It is fascinating how resilient bacteria can be, employing a diverse arsenal of genetic strategies to survive exposure to heavy metals like silver, a potent antimicrobial agent used for centuries. The development of silver resistance involves a complex interplay of mechanisms that go far beyond just a single efflux pump system like EmrAB. Indeed, the EmrAB-TolC pump has a broader role in multidrug resistance, and while it might be involved in general stress responses, specific and highly effective silver resistance is usually mediated by other, more specialized systems or a combination of mutations in various genes. Interestingly, in these silver nanoparticle-selected populations, a different component, part of the CusCFBA efflux system’s two-component sensor/responder system (specifically a regulator like CusS), is absent. Mutations in the cusS gene are a key mechanism for silver resistance in *E. coli*, allowing the Cus system to pump silver ions out via the CusCFBA efflux pump, often alongside other mutations (like in ompR) that reduce silver entry or work with the efflux system for a stronger defense [52]. Specific mutations, such as R15L or R438C, alter CusS’s function, enhancing its ability to sense silver and trigger the pump, leading to high tolerance, and these changes can be enhanced by interactions with other genes, showcasing complex adaptation [52]. Studies by Graves et al. [25] have demonstrated the involvement of CusS mutations in regulating intracellular silver concentrations, highlighting the complex interplay of various mechanisms in bacterial silver resistance.

Not surprisingly, other mutations occurred in green synthesized silver nanoparticles derived from reishi mushroom were involved in bacterial survival, growth, and interaction with their environment. These mutations were found in the following genes: DUF4756 family protein (*D1792_RS05680*), putative zinc-binding protein YnfU/cold shock-like protein (*ynfU/cspB*) and imcF-related family protein (*D1792_RS10035*). *D1792_RS05680* is considered essential for bacterial survival, suggesting they play crucial roles in their biology [53]. *ynfU/cspB* is often involved in various cellular processes, particularly those related to stress responses and gene regulation [54]. *D1792_RS10035* plays a significant role in bacterial virulence and interactions within their environment [55]. Understanding the functions of these mutations is crucial for several reasons. It reveals novel mechanisms and pathways involved in bacterial survival, growth, and interaction with their environment; they represent potential targets for the development of new antibiotics, and studying these mutations can offer insights into the evolution of protein function and the development of new biological processes.

Beyond the expected silver resistance mutations, among populations selected with reishi mushroom, green synthesized AgNPs also exhibited at least three other mutations, specifically impacting virulence and iron transport mechanisms. Mutations were observed in the catecholate siderophore receptor (*fiu*), catecholate siderophore esterase and invasion regulator (*sirB2*). *fiu* represent outer membrane proteins that facilitate the uptake of iron bound to catecholate siderophores [56]. These membrane protein receptors are crucial for bacterial survival, particularly in iron-limited environments, as they enable the bacteria to scavenge iron from their surroundings [57]. Catecholate siderophore receptors are considered virulence factors because they enable bacteria to acquire iron from the host, which is essential for their survival and proliferation during infection [58]. *IroE* is important for bacterial survival and virulence by allowing them to effectively utilize siderophores for iron acquisition and by enabling the activation of siderophore-based drug delivery systems [59]. In addition, *iroE* is required to activate siderophore–antibiotic conjugates, like Ent-Cipro, by hydrolyzing the siderophore component. This activation releases the antibiotic (e.g., ciprofloxacin) within the bacterial cell, leading to its antibacterial effect [60]. *sirB2* invasion controls the expression of genes involved in the process of bacterial invasion into host cells. These regulators ensure that invasion genes are activated at the right time and place, allowing bacteria to effectively penetrate host tissues [61].

Essentially, the human body employs a defense mechanism called nutritional immunity to deliberately restrict iron availability [62]. Iron is vital for bacteria to perform essential functions, such as replicating their DNA, generating energy through respiration, and carrying out general metabolic processes [63,64]. By locking away iron in host proteins like transferrin, ferritin, and hemoglobin, the body attempts to starve potential invaders and limit their growth [63,65]. In response to this iron-limited environment, pathogens have evolved highly effective survival mechanisms. They utilize sophisticated, high-affinity systems, most notably through the production of small molecules called siderophores, which act like molecular magnets to scavenge and “steal” iron from the host’s sequestered supplies [66].

Iron limitation enhances virulence and biofilm development, which protects bacteria from antibiotics and immune responses and promotes persistent infections [67,68]. Under conditions where iron is scarce, pathogenic bacteria, such as *Staphylococcus aureus*, activate specific genetic pathways to create protective communities known as biofilms. This biofilm formation is not a random response, but a highly regulated survival strategy designed to scavenge iron more effectively and resist the host’s defenses. A key player in this process is an iron-dependent regulator protein (like the Fur protein), which controls the genes necessary for producing the various components that make up the biofilm architecture [69]. Conversely, studies involving *Streptomyces coelicolor* and *Mycobacterium smegmatis* have shown that when iron is abundant, these bacteria can thrive and exhibit greater resistance to certain antibiotics [70]. Furthermore, by altering respiration and stress responses under varying iron conditions, bacteria can become significantly more tolerant to antibiotic treatments [71].

There are three specific genes where polymorphisms in green synthesized AgNP-resistant bacterial strains correlate with crucial functions related to iron metabolism and the ability to invade host tissues. Catecholate siderophore receptors (*fiu)* are outer membrane proteins that facilitate the uptake of iron bound to catecholate siderophores. These receptors are crucial for bacterial survival, particularly in iron-limited environments, as they enable bacteria to scavenge iron from their surroundings [57,58]. Catecholate siderophore receptors are considered virulence factors because they enable bacteria to acquire iron from the host, which is essential for their survival and proliferation during infection [58]. Catecholate siderophore esterase (*iroE*) is important for bacterial survival and virulence by allowing them to effectively utilize siderophores for iron acquisition and by enabling the activation of siderophore-based drug delivery systems [59]. In addition, *iroE* is required to activate siderophore–antibiotic conjugates, like Ent-Cipro, by hydrolyzing the siderophore component. This activation releases the antibiotic (e.g., ciprofloxacin) within the bacterial cell, leading to its antibacterial effect [60]. Invasion regulator *sirB2* controls the expression of genes involved in the process of bacterial invasion into host cells. These regulators ensure that invasion genes are activated at the right time and place, allowing bacteria to effectively penetrate host tissues [61].

After a 21-day exposure to green synthesized AgNPs from reishi mushrooms, bacteria were challenged with chemically synthesized AgNPs and exhibited an altered growth pattern, with growth being less robust than in control or untreated bacteria. This could often be due to phytochemical properties that alter their interaction with bacteria, making them a superior, less resistance-inducing alternative to chemical AgNPs. Furthermore, extracts in green synthesis significantly influence nanoparticle size and shape because their bioactive compounds act as both reducing and capping agents, controlling nucleation, growth, and preventing aggregation [11,72].

The green synthesis method in this study yielded a broader range of AgNP sizes and shapes compared to the more controlled chemical synthesis, suggesting that resistance mechanisms might be more effective against certain nanoparticle morphologies, thereby reducing their impact on differently structured nanoparticles. Green synthesized AgNPs were capped with biomolecules from the reishi mushroom (e.g., beta 1-3 glucans, ganoderic acid, and triterpenoids), while chemically synthesized AgNPs utilized different capping agents, potentially affecting their interaction with bacterial cells [11]. Furthermore, AgNPs’ stability and aggregation tendencies varied between synthesis methods, with resistance mechanisms possibly exploiting aggregation, which is more effective against less stable forms. In this study, chemically synthesized 20 nm AgNPs were stabilized in an aqueous citrate buffer to prevent aggregation, whereas green synthesized AgNPs were observed to aggregate.

Following 21 days of exposure to chemically synthesized AgNPs, a noticeable trend in population growth emerged. The population that underwent this selection process exhibited diminished growth compared to both the control group and the ancestral populations. Conversely, the ancestral populations demonstrated superior growth compared to both the control group and the selected population. This perplexing phenomenon might arise from the combined effects of stress caused by nanoparticles and how bacteria adapt to the stress. Chemical synthesized AgNPs exhibited antibacterial activity by generating reactive oxygen species (ROS), which damages cellular components, disrupts the cell membrane and interferes with metabolic pathways [73]. These multifaceted effects ultimately inhibit bacterial growth and can lead to cell death, underscoring the potential of nanoparticles as antibacterial agents. Also, the superior growth observed in the control populations, as compared to the treated populations, could be attributed to a specific mutation in the sugar kinase gene. Mutation in sugar kinase/hypothetical protein (D1792_RS06990/D1792_RS26615) enables the breakdown of sugars for energy production or their incorporation into cellular components [74].

Other possible explanations exist for why ancestral populations might exhibit greater growth in the presence of nanoparticles compared to populations that have been elected for 21 days. Resources invested in adapting to the selection pressure might be unavailable for developing resistance to the nanoparticles, which are known as evolutionary trade-offs [75]. Nanoparticles can have harmful effects on biological systems due to their small size, large surface area, and ability to interact with cellular components. In some cases, low concentrations of nanoparticles might have beneficial or growth-promoting effects on the ancestral populations (Hormesis-like effect). Unexposed populations might experience such a positive response, while the selected population might be past this point or have a different response due to prior adaptations. Furthermore, the ancestral population has not been subjected to nanoparticle exposure and, therefore, has not undergone selection for resistance to them, suggesting they have not incurred any potential fitness costs or trade-offs associated with such resistance, allowing for potentially higher overall growth in a nanoparticle-rich environment if the nanoparticles are not excessively toxic at the given concentration.

The chemically synthesized selected populations also showed superior 24 h growth relative to the controls and the ancestors in ionic silver. This can be linked to the selective sweeps in the EmrAB efflux pump, which acts as a sensor and switch, keeping the EmrAB efflux pump “off” when the environment is safe, and triggering its production when the cell faces exposure to noxious substances [51]. The absence of this mutation in the control and ancestral population could explain their poor growth in ionic silver. Other mutations unique to the chemically synthesized nanoparticle-selected population could have enhanced the growth performance in silver. For example, bacteria can employ various mechanisms to resist the oxidative stress caused by silver, and RNA polymerase sigma factor (*RpoE*), an extracytoplasmic function (ECF) sigma factor, appears to play a role in this resistance by initiating transcription by directing the RNA polymerase enzyme to specific DNA sequences [76]. RpoE is a key regulator of genes involved in adapting to various environmental stresses, including oxidative stress. When bacteria encounter silver, the resulting oxidative stress might trigger the RpoE pathway, leading to the activation of genes that help neutralize ROS or repair the damage they cause [77,78]. Furthermore, *RpoE* is involved in maintaining the integrity of the bacterial cell envelope, which is crucial against the damaging effects of silver [77]. By maintaining a healthy cell envelope, bacteria can reduce the influx of silver ions and mitigate the damage they cause [77,78]. While not explicitly mentioned in the context of silver and *RpoE*, *RpoE* has been linked to the induction of efflux pumps in some bacterial species, such as *Pseudomonas aeruginosa* [79]. These pumps can actively expel harmful substances, including metal ions, from the bacterial cell, potentially contributing to silver resistance. Energy associated with the efflux mechanism could be associated with mutations in other genes found in bacterial populations selected for chemically synthesized AgNPs; for example, succinate-CoA ligase subunit alpha (*sucD*) is essential for bacterial energy production and metabolism [80]. Acyl CoA:acetate/3-ketoacid CoA transferase (D1792_RS17175) facilitates the transfer of CoA from one molecule to another, enabling the utilization of various fatty acids and ketones as energy sources [81].

Energy and molecule transport in bacteria are intrinsically linked. Bacteria use metabolic energy to actively pull essential nutrients in and expel waste, while also generating energy from the transport of electrons and ions, often through complex membrane protein systems that couple energy generation to nutrient uptake, forming a vital cycle for survival and growth. In this study, selection in chemically synthesized AgNPs for 21 days resulted in mutation in a gene associated with transport; for example, intermembrane transport protein (*pqiB*) facilitates the movement of molecules between the inner and outer bacterial membranes, and across the periplasm. These proteins are crucial for various cellular processes, including nutrient uptake, protein secretion, and maintenance of cell envelope integrity [82].

Phage infection is a fundamental force in microbial ecology, controlling bacterial dynamics and evolution, while also presenting a promising, targeted solution to the global crisis of antibiotic resistance [83]. An important observation was that a particular selection process involving chemically synthesized AgNPs decreased the bacteria’s ability to develop resistance against phage attacks, as seen in mutation in retron system putative HNH endonuclease (*D1792_RS15055*). Retron system putative HNH endonuclease (*D1792_RS15055*) is known to play a role in bacterial defense against phage infection by acting as an effector protein [84].

One of the core observations in this study is that bacteria, when subjected to selective pressure from exposure to AgNPs created through environmentally friendly green synthesis methods, develop specific genomic changes associated with iron acquisition. Bacteria exposed to chemically synthesized AgNPs exhibited a similar characteristic. Exposure of a bacterial population to chemically synthesized AgNPs led to the selection of mutants with genomics alterations in the gene encoding the yersiniabactin polyketide synthase HMWP1 (also known as *Irp1*), a critical enzyme for siderophore production [85]. The core observation is that bacteria, when subjected to selective pressure from exposure to silver nanoparticles, whether those nanoparticles were created through environmentally friendly “green synthesis” methods or conventional “chemical synthesis” methods, develop specific genomic changes that cluster around a shared function, iron acquisition. Bacteria use siderophores to scavenge iron because it is vital for survival but scarce in most environments; these molecules act as powerful “iron grabbers,” allowing bacteria to compete, cause infections, promote motility/biofilm formation, and even influence host health, making them key to bacterial success, pathogen growth, and environmental cycling.

Bacterial life fundamentally depends on a tightly coupled relationship between energy generation and molecule transport. Bacteria utilize metabolic energy to actively import essential nutrients and expel waste products, concurrently generating energy through the movement of ions and electrons across specialized membrane protein systems. This cyclical process is vital for their growth and survival. This study highlights the vulnerability of this system to environmental factors. Exposure to chemically synthesized AgNPs over 21 days induced mutations in specific genes crucial for transport, such as the gene encoding the intermembrane transport protein (*pqiB*). *PqiB* proteins facilitate the vital movement of molecules across the bacterial cell envelope, specifically between the inner and outer membranes and across the periplasm [82]. Disruptions to these proteins impair critical cellular processes, including nutrient acquisition, protein secretion, and the maintenance of cell envelope integrity.

The interaction between bacteria and chemically synthesized AgNPs is more complex than initially understood. While researchers previously identified a specific mechanism involving the RpoE protein (a key regulator in stress responses) as a crucial player in bacterial survival against AgNPs, our studies have revealed that bacteria utilize several alternative strategies to overcome this metallic threat. The selected populations in chemically synthesized AgNPs showed mutations in two genes associated with stress and transport, for example, mutations in hypothetical protein (*D1792_RS20520*) and ClpP-like prohead protease/major capsid protein fusion protein (*D1792_RS03435*). Hypothetical protein (*D1792_RS20520*) is implicated in a wide range of cellular processes, including metabolism, and adaptation to various environments. ClpP-like prohead protease/major capsid protein fusion protein (*D1792_RS03435*) plays a significant role in bacterial stress responses such as oxidative stress [86]. Two-component system sensor histidine kinase (rcsC) detects environmental signals and initiates a cellular response, often via altered gene expression [87].

The chemically synthesized AgNP populations showed inferior 24 h growth relative to the controls in the green synthesized AgNPs from reishi mushroom. Several factors likely explain the reduced bacterial growth observed with chemically synthesized AgNPs, which could be due to the variations in stability, capping agent influence, size, and interaction with bacterial cells between green synthesized and chemically synthesized AgNPs, likely contributing to the observed differences in bacterial growth, as mentioned earlier. While the ancestral population showed superior growth in green synthesized AgNPs, its growth rate was unexpectedly lower than both the control and selected populations, highlighting a puzzling phenomenon that requires deeper examination. Smaller AgNPs produced via green synthesis often have a greater surface area compared to those produced through chemical methods. This larger surface area of green synthesized AgNPs can lead to increased interaction with bacterial cells [47]. Smaller nanoparticles readily penetrate bacterial membranes and cell walls, increasing their potential toxicity [88]. Control bacterial populations, grown in rich nutrient broth (NB) for 21 days, had a significant advantage over ancestral populations used in the experiments. This extended growth in nutrient-rich media allowed the control bacteria to build up reserves and potentially activate stress response mechanisms, repair cellular damage caused by nanoparticles, and maintain overall viability. In contrast, the ancestral populations were only cultured in NB for 24 h prior to the experiments, limiting their ability to benefit from the same level of nutrient abundance. Control populations exhibit increased growth compared to the ancestral population when exposed to green synthesized AgNPs. This enhanced growth can be attributed to selective sweeps occurring in specific genes within the control populations. Lipopolysaccharide core heptose (II)-phosphate phosphatase Ais/UDP-4-amino-4-deoxy-L-arabinose aminotransferase (*ais/arnB*) is crucial for bacterial resistance to antibiotics and plays a role in host defense mechanisms by modifying the lipid A component of lipopolysaccharide (LPS) [89]. Understanding these enzymes and their roles in LPS biosynthesis is critical for developing new approaches to combat antibiotic resistance and bacterial infections. DnaA initiator protein (*DnaA*) is a central element in bacterial DNA replication [90]. It orchestrates the initiation of replication at the origin of replication and influences gene expression, processes that can contribute to the ability to resist nanoparticles. The glutamine ABC transporter substrate-binding protein is a critical “grabber” protein essential for bacterial survival. It highly specifically binds to glutamine, a vital source of both nitrogen and carbon, and delivers it to the bacterial cell’s internal nutrient uptake system, thereby facilitating the cell’s ability to acquire these essential resources from its surroundings [91].

Other polymorphisms presented in both groups being studied (those exposed to chemically synthesized AgNPs and a control group) were relevant to fundamental biological functions. Quinolinate synthase (*nadA*) plays a crucial role in the biosynthesis of nicotinamide adenine dinucleotide (NAD+) [92]. Hypothetical protein (*D1792_RS20520*) is implicated in a wide range of cellular processes, including metabolism, virulence, and adaptation to various environments [93]. Propionate catabolism operon regulatory protein PrpR/methylisocitrate lyase (*prpR/prpB*) plays a crucial role in controlling the expression of genes [94]. Cyclic di-3′,5′-guanylate-activated glycosyltransferase (*nrfB*) plays a significant role in various cellular functions, particularly those related to surface adhesion, biofilm formation, and virulence [95].

The widespread environmental release of engineered nanomaterials drives microbial adaptations that accelerate the spread of multidrug-resistant infections. These nanoscale pollutants severely disrupt biogeochemical cycles and destabilize foundational food webs. Counteracting these compounding ecological threats necessitates nanoparticle-mediated bioremediation as a powerful weapon and applications of nanotechnology for green synthesis. These approaches rely on eco-friendly, biogenic nanomaterials that safely detoxify ecosystems without fostering further pathogenic resistance.

This study faces several notable limitations. While green synthesis is safer than chemical alternatives, it yields heterogeneous nanoparticles that complicate standardized toxicological evaluations. Additionally, the research neglects long-term effects and the potential for mutation-driven resistance. A lack of gene expression data also limits our understanding of molecular impacts, while omitting X-ray diffraction (XRD) restricts comprehensive characterization of the crystallite size, phase, and structure.

## 5. Conclusions

Nanoparticle fabrication falls into two main categories: conventional chemical methods (prioritizing precision with environmental drawbacks) and green synthesis methods (prioritizing sustainability using biological resources). This study reveals a significant and fascinating difference in the characterization of AgNPs from these distinct approaches, which in turn affects how *E. coli* bacteria develop resistance to them. Electron microscopy revealed that green synthesized AgNPs were heterogeneous in shape, unlike the uniformly spherical, chemically synthesized AgNPs. Despite these morphological differences, both types of AgNPs exhibited a strong absorption peak within the 440–450 nm range. It was found that over a 21-day period, *E. coli* rapidly developed robust resistance to “green synthesized” AgNPs (specifically those made using reishi mushrooms) and cross-resistance to traditional ionic silver, involving clear genomic and morphological adaptations. Intriguingly, this resistance to green synthesized AgNPs and ionic silver did not transfer to chemically synthesized AgNPs. In a contrasting result, the study observed that continuous exposure to the chemically synthesized AgNPs over the same 21-day period completely failed to induce a resistant phenotype in the bacterial population. Populations previously conditioned by exposure to chemically synthesized AgNPs exhibited an accelerated, superior, and more resilient growth response after 24 h when subsequently exposed to increasing concentrations of ionic silver due to mutation in genes associated with efflux. These outcomes are paramount, suggesting that chemically synthesized AgNPs present a much harder target for bacterial adaptation than green alternatives or ionic silver, and that the unique multi-target action of specific nanoparticles could be leveraged for designing next-generation antimicrobials that significantly impede the evolution of bacterial resistance. By understanding the specific ways in which bacteria attempt to resist nanoparticles and the potential for “cross-reactions” (like resistance to AgNPs conferring tolerance to ionic silver), researchers can optimize the design and synthesis of these powerful new tools in our ongoing fight against infectious diseases.

## Figures and Tables

**Table 1:** Genomic adaptation of populations selected by mushroom-synthesized silver nanoparticles over 21 days.

Gene	Position	Mutation	MNP1	MNP2	MNP3	MNP4	MNP5
*emrR*	1,917,559	W100 (TGG→TAG)	2 (0.75)	0	0	0	0
*diaA*	2,555,115	V98M (GTG→ATG)	1 (0.50)	0	1 (0.710)		1 (0.32)
*relA*	2,012,659	Q582K (CAA→AAA)	1 (0.47)	0	0	0	1 (0.35)
*D1792_RS05680*	1,078,228	A53T (GCC→ACC)	1 (0.42)	1 (0.46)	0	0	0
*D1792_RS06990/D1792_RS26615*	1,407,514	intergenic (+45/+58)	1 (0.36)	0	0	1 (0.35)	1 (0.31)
*uacT*	2,155,046	T314M (ACG→ATG)	1 (0.35)	0	0	0	0
*pstC/pstS*	3,187,389	intergenic (−58/+29)	1 (0.34)	0	0	0	0
*glnH*	4,869,651	A17V (GCG→GTG)	1 (0.34)	1 (0.37)	1 (0.42)	1 (0.36)	1 (0.45)
*ais/arnB*	1,526,714	intergenic (−240/−68)	1 (0.34)	3 (0.62)	0	0	2 (0.48)
*fiu*	4,862,171	T401P (ACC→CCC)	1 (0.33)	1 (0.31)	0	0	0
*ydjF*	877,943	K80K (AAG→AAA)	1 (0.30)	0	0	0	0
*iroE*	34,849	D45E (GAT→GAG)	1 (0.30)	0	0	1 (0.25)	0
*sirB2*	325,706	S9S (AGC→AGT)	1 (0.30)	0	0	0	0
*ynfU/cspB*	664,495	intergenic (+277/+87)	1 (0.28)	0	0	0	0
*baeS*	1,313,877	K2K (AAG→AAA)	1 (0.28)	0	0	1 (0.31)	0
*D1792_RS10035*	2,068,649	F759S (TTC→TCC)	1 (0.28)	1 (0.28)	0	0	0
*pdeN*	1,424,732	A163S (GCC→TCC)	1 (0.28)	0	0	0	0
*lolE*	151,100	T346P (ACC→CCC)	1 (0.27)	0	0	0	0
*dgoK*	3,151,724	A130V (GCT→GTT)	1 (0.27)	0	0	0	0
*xapA*	1,679,961	Y89S (TAC→TCC)	0	1 (0.46)	0	0	0
*lysO/aqpZ*	4,935,197	intergenic (−347/+147)	0	2 (0.46)	0	0	2 (0.46)
*yfhb*	1,835,793	L30F (TTA→TTC)	0	1 (0.42)	0	0	0
*D1792_RS02575*	461,348	E158E (GAG→GAA)	0	1 (0.40)	0	0	
*D1792_RS24525*	5,136,620	pseudogene (1217/1621 nt)	0	1 (0.39)	0	0	1 (0.35)
*sstT*	2,505,510	T264P (ACC→CCC)	0	1 (0.34)	0	1 (0.28)	1 (0.45)
*cysB/ymiA*	397,450	intergenic (+141/−178)	0	1 (0.34)	0	2 (0.58)	1 (0.31)
*D1792_RS14205*	2,922,928	L4L (TTA→CTA)	0	1 (0.34)	0	0	0
*D1792_RS20645/D1792_RS20650*	4,300,207	intergenic (−24/+319)	0	1 (0.32)	0	0	0
*rplJ*	3,488,354	A146T (GCT→ACT)	0	1 (0.28)	0	0	0
*mdtE*	2,923,441	A133T (GCA→ACA)	0	1 (0.28)	0	0	0
*cyoA*	4,525,732	N172N (AAC→AAT)	0	1 (0.28)	0	0	1 (0.31)
*cysJ/queD*	1,991,874	intergenic (−251/−68)	0	0	1 (0.40)	0	0
*sirB2*	325,706	S9S (AGC→AGT)	0	0	1 (0.38)	0	0
*D1792_RS02005*	348,466	L63 (TTA→TGA)	0	0	1 (0.35)	0	1 (0.38)
*D1792_RS02575*	461,348	E158E (GAG→GAA)	0	0	0	1 (0.34)	1 (0.38)
*pgaC*	5,086,604	I94I (ATA→ATC)	0	0	0	1 (0.25)	0
*gpJ*	641,105	E1049E (GAG→GAA)	0	0	0	1 (0.25)	0
*D1792_RS05680*	1,078,228	A53T (GCC→ACC)	0	0	0	1 (0.25)	1 (0.31)
*rplC*	2,709,134	P63P (CCT→CCG)	0	0	0	1 (0.23)	0
*fdrA*	4,616,036	I220T (ATT→ACT)	0	0	0	1 (0.23)	0
*fryC*	1,664,262	T163I (ACC→ATC)	0	0	0	1 (0.22)	0
*D1792_RS25790*	44,446	pseudogene (469/635 nt)	0	0	0	0	1 (0.41)
*D1792_RS06050*	1,201,539	pseudogene (1216/1620 nt)	0	0	0	0	1 (0.31)

Notes: After 21 days of selection with green synthesized AgNPs, five populations (MNP1-MNP5) were subjected to whole-genome resequencing. breseq 0.30 was used for sequence alignment and variant calling. The reported data includes the genes and mutations identified, along with their frequencies (in parentheses) and the number of times each mutation was observed (not in parentheses). Highlights in yellow denote instances where identical genetic polymorphisms were identified in every single experimental replicate performed. Genes in red are common in the control and other treatment populations. Genes highlighted red are present in the control population as well as all treatment populations.

**Table 2:** Description of genes.

Gene	Gene Product
*lysO/aqpZ*	L-lysine exporter LysO/aquaporin Z
*ais/arnB*	Lipopolysaccharide core heptose(II)-phosphate phosphatase Ais/UDP-4-amino-4-deoxy-L-arabinose aminotransferase
*emrR*	Multidrug efflux transporter EmrAB transcriptional repressor EmrR
*cysB/ymiA*	HTH-type transcriptional regulator CysB/YmiA family putative membrane protein
*cyoA*	Cytochrome o ubiquinol oxidase subunit II
*diaA*	DnaA initiator-associating protein DiaA
*sirB2*	Invasion regulator SirB2
*baeS*	Two-component system sensor histidine kinase BaeS
*D1792_RS15055*	Retron system putative HNH endonuclease
*D1792_RS06990/D1792_RS26615*	Sugar kinase/hypothetical protein
*glnH*	Glutamine ABC transporter substrate-binding protein GlnH
*D1792_RS02575*	AraC family transcriptional regulator
*thiB/sgrR*	Thiamine ABC transporter substrate binding subunit/DNA-binding transcriptional regulator SgrR
*pgaC*	Poly-beta-1,6-N-acetyl-D-glucosamine synthase
*uacT*	Urate/proton symporter UacT
*D1792_RS11095*	N-acetylneuraminate epimerase
*nrfB*	Cyclic di-3′,5′-guanylate-activated glycosyltransferase NrfB
*gpJ*	Phage attachment tail tip protein J
*rplJ*	50S ribosomal protein L10
*fiu*	Catecholate siderophore receptor Fiu
*mgtT/dgcZ*	Protein MgtT/diguanylate cyclase DgcZ
*fdrA*	Acyl-CoA synthetase FdrA
*D1792_RS05680*	DUF4756 family protein
*rplC*	50S ribosomal protein L3
*gltI/D1792_RS22555*	Glutamate/aspartate ABC transporter substrate-binding protein GltI/rhomboid family intramembrane serine protease
*irp1*	Yersiniabactin polyketide synthase HMWP1
*D1792_RS20520*	Hypothetical protein
*nadA*	Quinolinate synthase NadA
*D1792_RS20645/D1792_RS20650*	CPBP family intramembrane glutamic endopeptidase/hypothetical protein
*ybhR*	ABC transporter permease
*ydjF*	DeoR/GlpR family DNA-binding transcription regulator
*prpR/prpB*	Propionate catabolism operon regulatory protein PrpR/methylisocitrate lyase
*D1792_RS17175*	Acyl CoA:acetate/3-ketoacid CoA transferase
*rcsC*	Two-component system sensor histidine kinase RcsC
*mdoG*	Glucans biosynthesis protein MdoG
*pqiB*	Intermembrane transport protein PqiB
*nrfB*	Cyclic di-3′,5′-guanylate-activated glycosyltransferase NrfB
*D1792_RS15055*	Retron system putative HNH endonuclease
*relA*	GTP diphosphokinase
*D1792_RS02005*	Phosphatase PAP2 family protein
*D1792_RS25790*	Hypothetical protein
*D1792_RS02575*	AraC family transcriptional regulator
*rpoE*	RNA polymerase sigma factor RpoE
*D1792_RS03435*	ClpP-like prohead protease/major capsid protein fusion protein
*xapA*	Xanthosine phosphorylase
*yfhb*	Phosphatidylglycerophosphatase C
*ycdZ*	DUF1097 domain-containing protein
*rplJ*	50S ribosomal protein L10
*nrdH*	Glutaredoxin-like protein NrdH
*sucD*	Succinate-CoA ligase subunit alpha
*atpG*	F0F1 ATP synthase subunit gamma
*ulaG*	L-ascorbate 6-phosphate lactonase
*D1792_RS03435*	ClpP-like prohead protease/major capsid protein fusion protein
*bfd/chiA*	Bacterioferritin-associated ferredoxin/bifunctional chitinase/lysozyme
*yjcE/D1792_RS17315*	Na+/H+ antiporter/LysR family transcriptional regulator
*pstC/pstS*	Phosphate ABC transporter permease PstC/phosphate ABC transporter substrate-binding protein PstS
*D1792_RS24525*	ISL3 family transposase
*rhaD*	Rhamnulose-1-phosphate aldolase
*gnsB*	GnsA/GnsB family addiction module toxin
*fryC*	PTS fructose transporter subunit IIC
*sstT*	Serine/threonine transporter SstT
*rnb*	Exoribonuclease II
*pic/D1792_RS20755*	Serine protease autotransporter toxin Pic/hypothetical protein
*pstC/pstS*	Phosphate ABC transporter permease PstC/phosphate ABC transporter substrate-binding protein PstS
*iroE*	Catecholate siderophore esterase IroE
*ynfU/cspB*	Putative zinc-binding protein YnfU/cold shock-like protein CspB
*dgoK*	2-dehydro-3-deoxygalactonokinase
*D1792_RS10035*	ImcF-related family protein
*pdeN*	Cyclic di-GMP phosphodiesterase
*lolE*	Lipoprotein-releasing ABC transporter permease subunit LolE
*D1792_RS14205*	Hypothetical protein
*cysB/ymiA*	HTH-type transcriptional regulator CysB/YmiA family putative membrane protein
*D1792_RS02005*	Phosphatase PAP2 family protein
*D1792_RS06050*	ISL3 family transposase

**Table 3:** Analysis of genomic adaptations observed in populations selected by exposure to chemically synthesized silver nanoparticles after 21 days.

Gene	Position	Mutation	CNP1	CNP2	CNP3	CNP4	CNP5
*diaA*	2,555,115	V98M (GTG→ATG)	1 (0.47)	1 (0.37)	1 (0.36)	1 (0.39)	1 (0.39)
*glnH*	4,869,651	A17V (GCG→GTG)	1 (0.46)	1 (0.40)	1 (0.31)	2 (0.26)	1 (0.28)
*nadA*	4,803,678	V43G (GTG→GGG)	1 (0.46)	0	2 (0.44)	0	0
*uacT*	2,155,046	T314M (ACG→ATG)	1 (0.38)	0	0	0	1 (0.25)
*cyoA*	4,525,732	N172N (AAC→AAT)	1 (0.37)	1 (0.32)	1 (0.33)	1 (0.35)	1 (0.42)
*yfhb*	1,835,844	L13F (TTA→TTC)	2 (0.36)	0	0	0	0
*ycdZ*	73,234	Y69S (TAC→TCC)	1 (0.35)	0	0	0	0
*gpJ*	641,105	E1049E (GAG→GAA)	1 (0.33)	1 (0.30)	1 (0.26)	2 (0.45)	0
*mdoG*	82,367	A100T (GCC→ACC)	1 (0.30)	0	0	0	0
*rplJ*	3,488,354	A146T (GCT→ACT)	1 (0.30)	0	0	1 (0.35)	0
*D1792_RS20520*	4,275,435	T20P (ACT→CCT)	2 (0.29)	0	0	1 (0.24)	0
*nrdH*	1,907,242	Q13R (CAG→CGG)	1 (0.28)	0	0	0	0
*sucD*	4,790,571	A218T (GCA→ACA)	1 (0.28)	0	0	0	0
*sirB2*	325,706	S9S (AGC→AGT)	1 (0.28)	0	1 (0.35)	0	1 (0.32)
*baeS*	1,313,877	K2K (AAG→AAA)	1 (0.27)	0	1 (0.28)	0	1 (0.32)
*atpG*	3,194,348	A284V (GCC→GTC)	1 (0.27)	0	0	0	0
*emrR*	1,917,423	E55K (GAG→AAG)	1 (0.27)	1 (0.35)	0	0	0
*D1792_RS02005*	348,445	S56L (TCG→TTG)	1 (0.27)	1 (0.38)	0	0	4 (0.53)
*ais/arnB*	1,526,719	intergenic (−245/−63)	0	3 (0.44)	2 (0.53)	0	0
*lysO/aqpZ*	4,935,190	intergenic (−340/+154)	0	1 (0.42)	0	0	1 (0.58)
*relA*	2,012,659	Q582K (CAA→AAA)	0	1 (0.39)	0	0	0
*ybhR*	4,847,202	M292L (ATG→CTG)	0	1 (0.37)	1 (0.37)	0	0
*rplC*	2,709,161	A54A (GCT→GCG)	0	1 (0.37)	0	1 (0.24)	0
*D1792_RS25790*	44,446	pseudogene (469/635 nt)	0	1 (0.36)	0	0	0
*D1792_RS02575*	461,348	E158E (GAG→GAA)	0	1 (0.36)	0	0	1 (0.45)
*cysB/ymiA*	397,446	intergenic (+137/−182)	0	1 (0.34)	0	0	0
*rpoE*	1,847,229	R39H (CGC→CAC)	0	1 (0.32)	0	0	0
*D1792_RS03435*	653,545	D456E (GAT→GAG)	0	1 (0.32)	0	0	0
*rcsC*	1,479,289	D638Y (GAT→TAT)	0	1 (0.31)	1 (0.29)	0	0
*xapA*	1,679,961	Y89S (TAC→TCC)	0	1 (0.30)	0	0	0
*D1792_RS20645/* *D1792_RS20650*	4,300,207	intergenic (−24/+319)	0	1 (0.40)	0	0	0
*ydjF*	877,943	K80K (AAG→AAA)	0	0	1 (0.36)	0	0
*nadA*	4,803,680	M44V (ATG→GTG)	0	0	1 (0.35)	0	0
*prpR/prpB*	4,437,586	intergenic (−33/−206)	0	0	1 (0.32)	0	0
*D1792_RS17175*	3,575,875	A202T (GCT→ACT)	0	0	1 (0.31)	0	0
*pqiB*	5,022,977	L28F (CTC→TTC)	0	0	1 (0.26)	0	0
*nrfB*	4,635,451	N431N (AAC→AAT)	0	0	1 (0.25)	0	1 (0.22)
*D1792_RS15055*	3,115,863	R2R (AGG→AGA)	0	0	1 (0.25)	0	1 (0.30)
*fiu*	4,862,171	T401P (ACC→CCC)	0	0	0	1 (0.31)	0
*mgtT/dgcZ*	625,697	intergenic (+34/+81)	0	0	0	1 (0.30)	0
*fdrA*	4,616,036	I220T (ATT→ACT)	0	0	0	1 (0.28)	0
*D1792_RS05680*	1,078,228	A53T (GCC→ACC)	0	0	0	1 (0.28)	0
*gltI/D1792_RS22555*	4,719,927	intergenic (−126/−328)	0	0	0	1 (0.26)	0
*irp1*	1,100,609	D635E (GAC→GAA)	0	0	0	1 (0.24)	0
*D1792_RS06990/* *D1792_RS26615*	1,407,514	intergenic (+45/+58)	0	0	0	0	1 (0.28)
*thiB/sgrR*	4,087,057	intergenic (−144/+20)	0	0	0	0	1 (0.26)
*pgaC*	5,086,604	I94I (ATA→ATC)	0	0	0	0	1 (0.26)
*D1792_RS11095*	2,296,923	G343G (GGG→GGT)	0	0	0	0	1 (0.23)

Notes: After 21 days of selection with chemically synthesized AgNPs, five populations (CNP1-CNP5) were subjected to whole-genome resequencing. breseq 0.30 was used for sequence alignment and variant calling. The reported data includes the genes and mutations identified, along with their frequencies (in parentheses) and the number of times each mutation was observed (not in parentheses). Highlights in yellow denote instances where identical genetic polymorphisms were identified in every experimental replicate performed. Genes highlighted red are present in the control population as well as all of the treatment populations.

**Table 4:** Control populations’ single-nucleotide polymorphisms at day 21.

Gene	Position	Mutation	C1	C2	C3	C4	C5
*lysO/aqpZ*	4,935,197	intergenic (−347/+147)	3 (0.73)	0	0	2 (0.70)	0
*ais/arnB*	1,526,714	intergenic (−240/−68)	1 (0.34)	3 (0.45)	3 (0.46)	1 (0.35)	3 (0.36)
*cysB/ymiA*	397,446	intergenic (+137/−182)	1 (0.32)	1 (0.31)	1 (0.31)	0	1 (0.34)
*relA*	2,012,659	Q582K (CAA→AAA)	1 (0.3)	1 (0.34)	1 (0.34)	1 (0.33)	1 (0.30)
*ulaG*	3,750,485	F214L (TTC→TTA)	1 (0.3)	0	0	0	0
*D1792_RS03435*	653,545	D456E (GAT→GAG)	1 (0.29)	0	0	1 (0.30)	0
*glnH*	4,869,651	A17V (GCG→GTG)	1 (0.29)	1 (0.46)	1 (0.46)	1 (0.27)	1 (0.45)
*uacT*	2,155,046	T314M (ACG→ATG)	1 (0.28)	0	0	1 (0.50)	0
*D1792_RS01330*	230,889	Q38K (CAG→AAG)	1 (0.27)	0	0	0	0
*bfd/chiA*	2,723,549	intergenic (−159/+10)	1 (0.26)	0	0	0	0
*rplJ*	3,488,354	A146T (GCT→ACT)	1 (0.25)	1 (0.25)	1 (0.25)	0	0
*D1792_RS20520*	4,275,429	T22P (ACC→CCC)	1 (0.25)	0	0	0	0
*prpR/prpB*	4,437,586	intergenic (−33/−206)	1 (0.25)	1 (0.34)	1 (0.34)	1 (0.38)	0
*diaA*	2,555,115	V98M (GTG→ATG)	1 (0.25)	1 (0.39)	1 (0.39)	1 (0.37)	1 (0.48)
*yjcE/D1792_RS17315*	3,609,987	intergenic (+6/+30)	1 (0.24)	0	0	0	0
*nadA*	4,803,681	M44R (ATG→AGG)	1 (0.23)	0	0	0	0
*mdoG*	82,367	A100T (GCC→ACC)	1 (0.22)	1 (0.39)	1 (0.31)	0	1 (0.30)
*pstC/pstS*	3,187,389	intergenic (−58/+29)	1 (0.47)	0	1 (0.41)	0	1 (0.41)
*D1792_RS24525*	5,136,620	pseudogene (1217/1621 nt)	1 (0.40)	0	1 (0.40)	1 (0.47)	0
*D1792_RS02575*	461,348	E158E (GAG→GAA)	1 (0.39)	0	1 (0.39)	0	0
*xapA*	1,679,961	Y89S (TAC→TCC)	1 (0.36)	0	1 (0.36)	0	0
*rhaD*	3,389,307	V138G (GTG→GGG)	1 (0.32)	0	1 (0.32)	0	1 (0.32)
*gnsB*	660,927	V33I (GTT→ATT)	1 (0.31)	0	1 (0.31)	0	0
*fryC*	1,664,262	T163I (ACC→ATC)	1 (0.29)	0	1 (0.29)	0	0
*ydjF*	877,943	K80K (AAG→AAA)	1 (0.29)	0	1 (0.29)	1 (0.35)	0
*cyoA*	4,525,732	N172N (AAC→AAT)	1 (0.28)	0	1 (0.28)	0	1 (0.33)
*pgaC*	5,086,604	I94I (ATA→ATC)	1 (0.25)	0	1 (0.25)	1 (0.26)	0
*baeS*	1,313,877	K2K (AAG→AAA)	0	0	0	1 (0.37)	1 (0.31)
*sstT*	2,505,510	T264P (ACC→CCC)	0	0	0	1 (0.32)	0
*rnb*	410,541	V278I (GTC→ATC)	0	0	0	1 (0.31)	0
*gpJ*	641,105	E1049E (GAG→GAA)	0	0	0	1 (0.31)	0
*D1792_RS20645/D1792_RS20650*	4,300,207	intergenic (−24/+319)	0	0	0	2 (0.25)	0
*nrfB*	4,635,451	N431N (AAC→AAT)	0	0	0	1 (0.24)	0
*D1792_RS02005*	348,466	L63 (TTA→TGA)	0	0	0	0	1 (0.38)
*D1792_RS06050*	1,201,539	pseudogene (1216/1620 nt)	0	0	0	0	1 (0.36)
*D1792_RS11890*	2,466,313	R393R (CGT→CGG)	0	0	0	0	1 (0.36)
*fdrA*	4,616,036	I220T (ATT→ACT)	0	0	0	0	1 (0.33)
*pic/D1792_RS20755*	4,333,278	intergenic (−1076/−30)	0	0	0	0	3 (0.31)

Notes: After 21 days of selection with green synthesized AgNPs, five populations (C1–C5) were of the control subjected to whole-genome resequencing. breseq 0.30 was used for sequence alignment and variant calling. The reported data includes the genes and mutations identified, along with their frequencies (in parentheses) and the number of times each mutation was observed (not in parentheses). Highlights in yellow denote instances where identical genetic polymorphisms were identified in every experimental replicate performed. Genes highlighted red are present in the control population as well as all of the treatment populations.

## Data Availability

Data are contained within the article.

## Supplementary Materials

The following supporting information can be downloaded at https://www.mdpi.com/article/10.3390/nano16120730/s1, Figure S1: Zeta potential distribution of Ganoderma lucidum (Reishi)-mediated silver nanoparticles (MNPs) (green synthesized AgNPs); Figure S2: FTIR analysis of Ganoderma lucidum (Reishi) extract and synthesized AgNPs (green synthesized AgNPs); Figure S3: FTIR analysis of *Ganoderma lucidum* (Reishi) extract.
