# Supplementary material for: Bacterial Adaptive Responses to Green and Chemically Synthesized Silver Nanoparticles: Implications for Resistance Development
Source: Nanomaterials (Basel). 2026 Jun 12;16(12):730. doi: 10.3390/nano16120730 (PMC13306010; doi:10.3390/nano16120730)
Supplement: Supplementary file 1 [file nanomaterials-16-00730-s001.zip › nanomaterials-4339910-supplementary.pdf]

Supplementary Materials

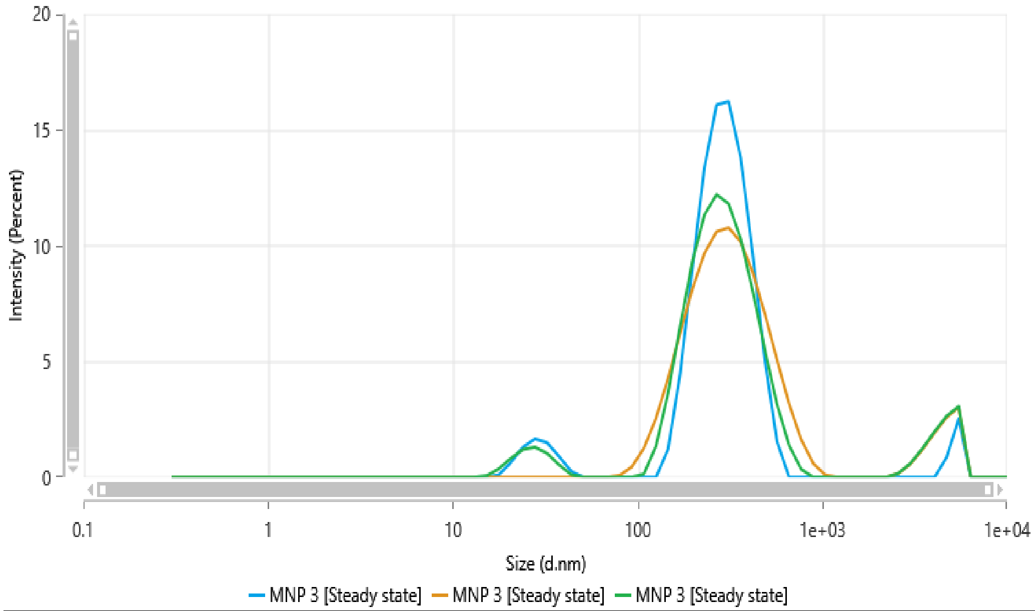

Name	Mean	Standard Deviation	RSD	Minimum	Maximum
Zeta Potential (mV)	-25.76	0.6472	2.512	-26.32	-25.05
Conductivity (mS/cm)	0.326	0	0	0.326	0.326
Wall Zeta Potential (mV)	-22.8	3.016	13.23	-26.23	-20.58
Quality Factor	3.483	0.4153	11.92	3.032	3.848
Zeta Peak 1 Mean (mV)	-25.76	0.6472	2.512	-26.32	-25.05

Figure S1. Zeta potential distribution of *Ganoderma lucidum* (Reishi)-mediated silver nanoparticles (MNPs) (green synthesized AgNPs). A surface charge of -25.76mV indicates high colloidal stability.

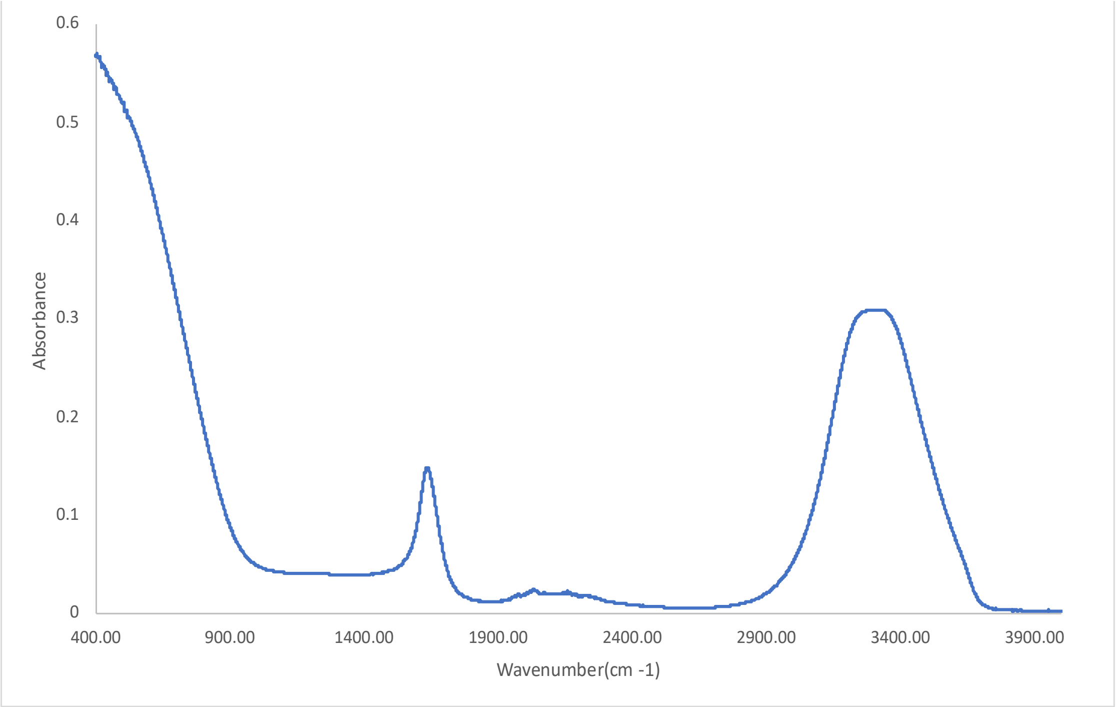

Figure S2. FTIR analysis of *Ganoderma lucidum* (Reishi) extract and synthesized AgNPs (green synthesized AgNPs). The profiles highlight the specific biomolecular functional groups acting as reducing, capping, and stabilizing agents during nanoparticle formation.

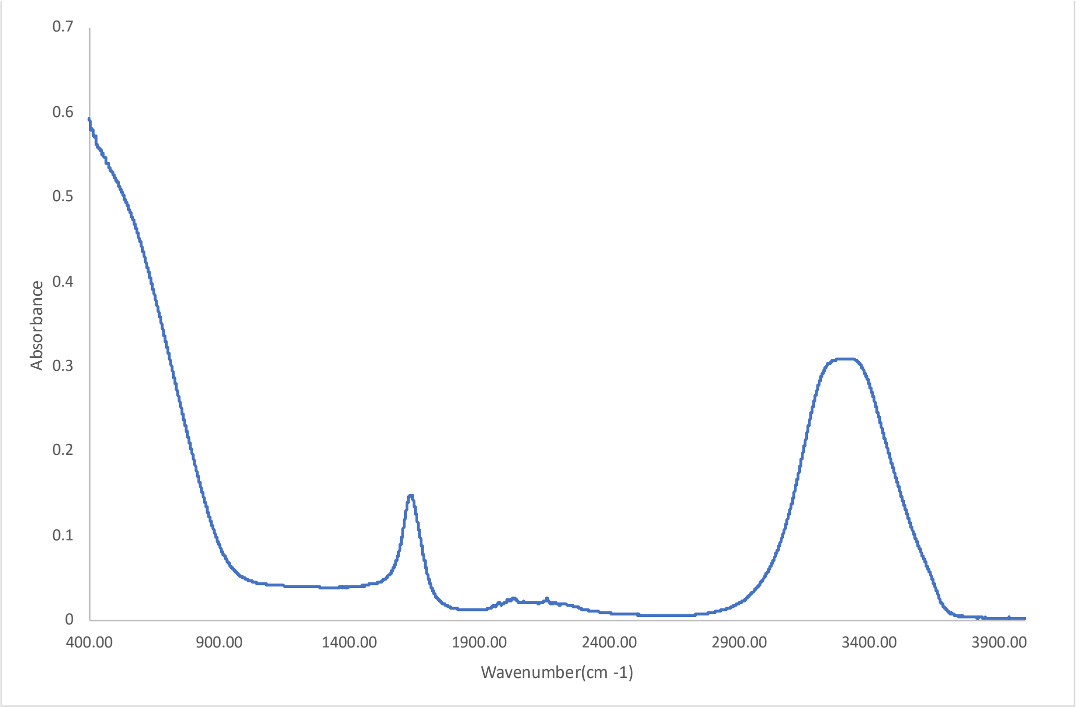

Figure S3. FTIR analysis of *Ganoderma lucidum* (Reishi) extract. The profiles highlight the specific biomolecular functional groups acting as reducing, capping, and stabilizing agents during nanoparticle formation.
